# Humidity drives spontaneous OH oxidation of organic particles

**DOI:** 10.1126/sciadv.adx4507

**Published:** 2025-06-20

**Authors:** Maria Angelaki, D. James Donaldson, Sébastien Perrier, Matthieu Riva, Christian George

**Affiliations:** ^1^ Universite Claude Bernard Lyon 1, CNRS, IRCELYON, UMR 5256, Villeurbanne F-69100, France.; ^2^ Department of Chemistry, University of Toronto, 80 George Street, Toronto, ON M5S 3H6, Canada.; ^3^ Department of Physical and Environmental Sciences, University of Toronto, Scarborough, 1265 Military Trail, Toronto, ON M1C 1A4, Canada.; ^4^ Tofwerk AG, Thun, Bern, Switzerland.

## Abstract

We report evidence that organic aerosols containing carboxylic acids can be spontaneously oxidized in the dark under normal atmospheric conditions due to interfacial hydroxyl radical production. Product formation is negligible under dry conditions and increases with increasing relative humidity. In a dioxygen-free environment, the oxidation efficiency is substantially decreased. Size-resolved measurements show an increase in the reactivity and product formation yields for smaller particles, correlated with their surface-to-volume ratio. Our findings suggest that spontaneous hydroxyl radical production at the air-water interface of organic nanodroplets may be an important pathway in their oxidation, especially during nighttime.

## Teaser

Spontaneous interfacial OH production leads to substantial organic aerosol aging, especially during nighttime.

## INTRODUCTION

Organic aerosols (OAs) are ubiquitous in the atmosphere and are of utmost importance for climate, air quality, and human health *1*–*3*). Atmospheric OA particles participate in important heterogeneous reactions, both on their surfaces and in their bulk phase (*4*). During their lifetime in the atmosphere, organic particles can undergo multiphase reactions with gas-phase tropospheric oxidants, such as hydroxyl radicals (OH), nitrate radicals (NO_3_), and ozone (O_3_), altering their chemical composition and atmospheric impact, on both regional and global scales (*5*–*8*).

Considerations of OH radical reactions in the atmosphere have mainly involved daytime chemistry due to the sunlight-driven formation of OH(g) in the troposphere (*9*). Very recently, several studies have reported evidence that OH radicals can be spontaneously produced near the air-water interface of aqueous droplets, without the necessity of any catalyst or light (*10*–*13*). For example, Li *et al.* (*12*) used organic compounds [i.e., terephthalic acid (TA)] as an OH trap, and they demonstrated OH formation by detecting 2-hydroxyterephthalic acid, which is a known product of the reaction of OH radicals with TA. Other groups demonstrated this process, via monitoring the production of H_2_O_2_, performing both direct and indirect measurements (*10*, *13*–*17*). It has been suggested that the reason for OH generation near the air-water interface is that hydroxide ions (OH^−^) undergo charge separation at the interface, due to the presence of a strong interfacial electric field (*18*–*20*), leading to the formation of OH radicals and solvated electrons (*10*, *12*, *14*, *21*). Other groups have attributed the phenomenon to the difference in the solvation energies of the ions at this interface and bulk, which results to changes in the interfacial reduction potential (*10*, *15*).

**Fig. 1.**
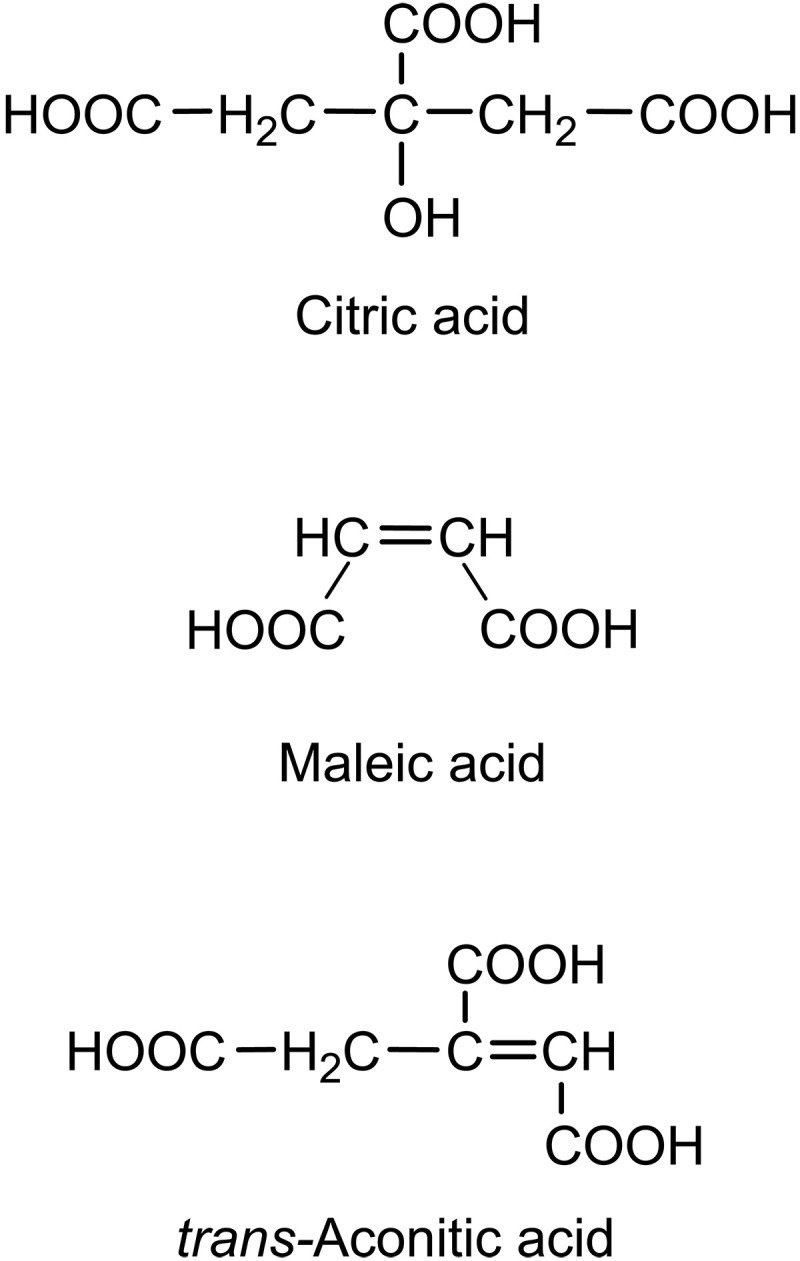
Chemical structures of citric acid (CA), maleic acid (MA), and *trans-*Aconitic acid (*trans*-AA).

In this work, we investigated the oxidation of laboratory-generated OAs in humidified air in the absence of any OH radical precursors or light. Citric acid (CA), maleic acid (MA), and *trans*-aconitic acid (AA) were selected for this study due to their atmospheric abundance and for their physicochemical properties (high reactivity and low vapor pressure) (*22*–*25*). The chemical structures of the organic compounds are given in Fig. 1. Aqueous solutions of these acids were nebulized, and the polydisperse particles were dried and introduced into a flow tube reactor (fig. S1). The humidity inside the reactor was set between 0 and 99%, regulated by dry or humidified, purified compressed air. Particles and water vapor interact for a reaction time of 90 s. Last, the particles were collected onto filters, and their chemical composition was determined and quantified by means of liquid chromatography coupled with high-resolution mass spectrometry (LC-HRMS) (text S1 and fig. S2).

## RESULTS

### Formation of OH oxidation products in organic nanoparticles at different humidity levels

**Fig. 2.**
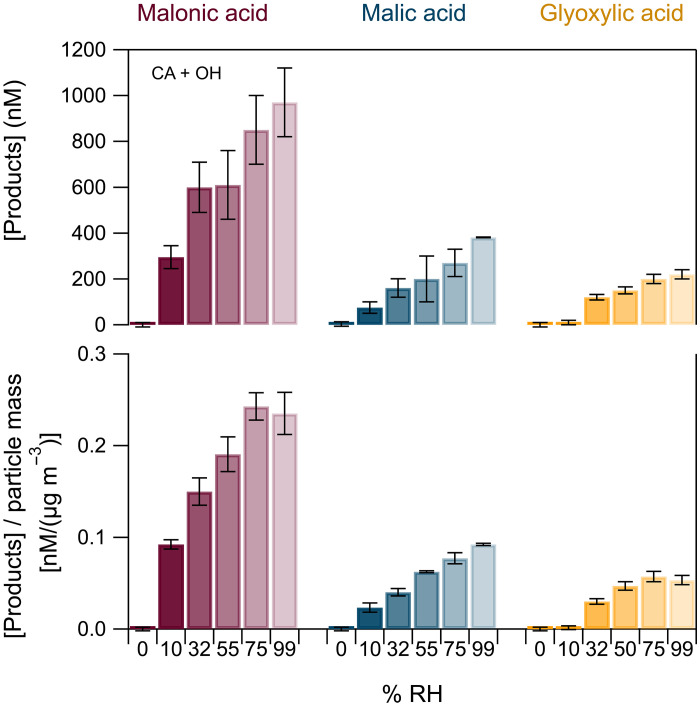
Products formation at various humidity levels. (**Top**) Product concentrations as a function of RH, when CA particles were exposed to increasing levels of humidity. The RH increases as the color scale fades. (**Bottom**) Product concentration normalized by the measured total particle mass, also as a function of RH.

Figure 2 (top) (and tables S1 and S4) displays the effect of relative humidity (RH), between 0 and 99%, on the concentrations of products formed in CA particles. The structures of the products are given in fig. S3. The results using MA and *trans*-AA droplets are similar and presented in text S2 (figs. S4 to S6) and tables S2, S3, S5, and S6.

Under dry conditions (RH = 0%), no oxidation products were detected. However, already at very low humidity (10% RH), CA was oxidized, and malonic, malic, and glyoxylic acids were observed and quantified as products. Those are the products expected from the OH oxidation of CA (*26*). Product concentrations show a systematic increase as a function of increasing humidity, suggesting that, at higher water content, OH radical production is promoted. To ensure that product formation is associated exclusively with the particle phase, we also analyzed corresponding bulk solutions. None of these products was observed in the bulk solution (text S2). To eliminate any possible contribution of light-induced processes, we carried out all experiments under dark conditions (including those in bulk solution). Last, to represent better the complex composition of atmospheric particles, we performed flow tube experiments at 0 and 55% of RH, where bulk solutions containing a mixture of CA, MA, and AA were used rather than the single-component acid particles. Under dry conditions, no products were observed; in the presence of water vapor, we find that the concentrations of reaction products represent the overall concentration obtained by spraying separately each organic compound. These findings suggest that the interaction of several organics within one particle does not affect their oxidation (fig. S7).

The organic acid particles are hygroscopic and will gradually take up water at elevated RH, resulting in their growth (*27*–*29*). The water uptake increases abruptly after the deliquescence point, which is >70%, for all cases (*30*–*32*). Product formation starts before the particle deliquesces, which is the first indication that suggests that chemistry is happening on the surfaces. The particle size was observed to increase with the increase in water content inside the reactor due to uptake of water (text S3 and figs. S8 to S10). To verify that the increase in product concentrations with increasing RH is not merely a consequence of increasing the CA particle size, we normalized the product concentrations to the total particle mass concentration (Fig. 2, bottom). In the Supplementary Materials, we also give the concentrations normalized by the total particle surface area (figs. S4 to S6). These normalized concentrations display the same trend as that of the absolute concentrations, confirming that product formation is greatly favored at high levels of humidity. No substantial differences in the product concentrations were observed above 75% RH; for malonic and glyoxylic acids, the normalized concentrations may even display a slight decrease. At RH values above the deliquescence point, the particles are liquid droplets, and, therefore, we attribute the decrease in normalized product amounts as likely due to dilution by the high amount of water inside the droplets.

### The effect of particle size on the product yields

The dependence of reactivity on particle size gives important insights into the relative role of surface chemistry in the reactions of interest (*33*–*37*). To investigate how particle size affects the spontaneous oxidation of the organic droplets, we performed size-resolved measurements at a fixed RH value of 55% (text S4). Specific sizes of dry polydisperse organic particles were selected on the basis of particle aerodynamic diameter (Da), using an aerodynamic aerosol classifier (AAC) before their entrance to the flow tube reactor. At the reactor outlet, the particle size distributions were recorded to monitor their growth during the reaction time, based on their electrical mobility diameter (Dm). The size measurements are described and displayed in the Supplementary Materials (text S4 and figs. S11 to S14). Last, the particles were collected and analyzed offline, using LC-HRMS. We quantified the product yield formation by dividing the concentration of each product by the concentration of the reactant (text S4, fig. S15, and tables S7 to S9).

**Fig. 3.**
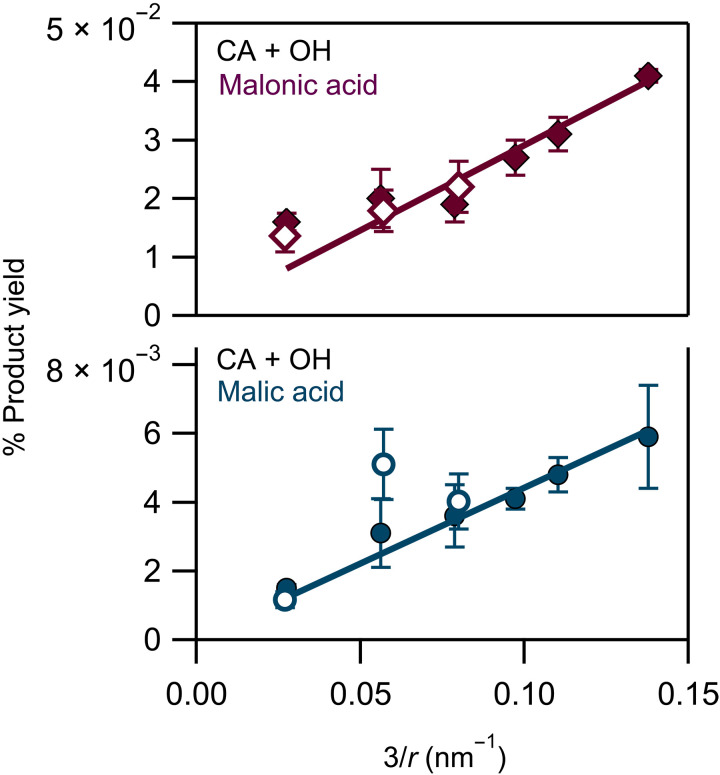
Product yields at various particle sizes. Malonic acid (red diamonds) and malic acid (blue circles) formation yields, quantified by liquid chromatography–mass spectrometry, as a function of the particle surface-to-volume ratio [3/radius (*r*)] in CA. Solid and open symbols represent measurements performed using AAC and DMA, respectively. The radius is calculated on the basis of the electrical Dm. Products at 3/*r* higher than 0.08 were not detectable using DMA due to the low total mass.

In Fig. 3, the formation yields (%) of the major products of the OH reaction with CA are presented as a function of the particle surface-to-volume ratio. Only malonic and malic acids were detected during these offline monodisperse measurements; glyoxylic acid was not observed because of the fact that its concentration was below the limit of quantitation. The product yields increased linearly as a function of the particle surface-to-volume ratio, suggesting that the reaction occurs at the interface and it is favored in smaller droplets, i.e., high surface-to-volume ratio. Similar to what we see in CA, the yields of the products formed in MA and *trans*-AA particles also increase in smaller particles; these results are given in the Supplementary Materials (fig. S15). Overall, MA oxidation let to higher product concentrations (fig. S5) and formation yields (fig. S15), as its reaction rate with OH is two orders of magnitude greater than that of CA (*k*_MA+OH_ = 6 × 10^9^ M^−1^ s^−1^ and *k*_CA+OH_ = 5 × 10^7^ M^−1^ s^−1^) (*38*, *39*). To the best of our knowledge, there is no kinetic data available for the reaction of *trans*-AA with OH radicals.

The presence of charges within the particles has been suggested as one potential key driver in interfacial OH production, and this is a still controversial subject (*40*, *41*). While our particles may carry a small number of charges, they never reach a Coulomb explosion regime, as their size is constant during their flight time (they are not evaporating). To investigate the charge effect, we induced additional charges on the particles during size-selected experiments for CA particles, using a differential mobility analyzer (DMA) connected to an x-ray neutralizer. With this technique, particles that enter the system experience changes in their natural charges induced by the neutralizer; this was verified by the recorded CA size distributions after their reaction, where particles appeared, which displayed changes in charge state of ±1 (text S4 and fig. S16). The product yield data obtained using the DMA are in good agreement with those selected by means of an AAC, within our uncertainties and the limits of our detection, suggesting that changes in the charge state of the particles have a negligible effect on the spontaneous oxidation of the organic droplets studied here (Fig. 3, open symbols).

Online measurements were also performed using the particle-phase WALL-E (wall-free particle evaporator) inlet coupled with a Vocus adduct ionization mechanism (AIM) high-resolution mass spectrometer (*42*, *43*), aiming to achieve measurements of high sensitivity (text S5, figs. S17 to S25, and tables S1 to S3). The product yields of malonic and malic acids using CA particles at 55% RH are measured to be higher in smaller droplets, in agreement with the offline measurements. Note that these results are only qualitative because of the lack of calibration factors. During the online measurements with CA, additional products, such as C_5_H_6_O_5_ (oxyglutaric acid) and C_5_H_6_O_6_ were observed, with their formation yields higher in smaller droplets. This demonstrates the added value of using complementary techniques for the complete particle-phase chemical characterization. The formation of these compounds is consistent with what is reported in previous studies (*26*); they were not detected with the offline analysis most probably due to their decomposition in the heated electrospray ionization source (HESI) and/or their low concentrations that may be below the detection limit of the instrument.

### Mechanistic investigation

The results presented above provide strong evidence that OH radicals are formed near the interface of organic particles and initiate oxidation of the carboxylic acids. To understand the formation of the major identified products, we suggest the following OH-induced oxidation pathways, which are described in detail in text S6 and figs. S26 to S28.

The OH radical reaction with CA proceeds via hydrogen (H) abstraction from the ─CH_2_ group and/or one of the carboxylic groups (fig. S26, A to C) (*44*). We performed density functional theory calculations at the ωB97X-V/TZVPPD level to calculate the energetics of the three possible reaction pathways. Hydrogen abstraction of the middle (P1) and side (P2) ─COOH groups is exoergic by −126 and −79 kJ mol^−1^, respectively, followed by spontaneous decarboxylation. Over 40 years ago, Corvaja *et al.* (*44*) also reported the decarboxylation of CA during reaction with OH radical. To the best of our knowledge, the energetics of this process are presented here. A future publication from our group will explore the reactive decarboxylation mechanism more fully. Hydrogen abstraction of the ─CH_2_ group (P3) is also thermodynamically favored, by −80 kJ mol^−1^.

In pathway P1, the initially formed radicals (**·**R) react with O_2_ to form oxyglutaric, malonic, and glyoxylic acids, via a sequence which also involves RO_2_ reactions (fig. S26A). In a similar manner, glyoxylic acid and C_5_H_6_O_6_ are formed via P2 (fig. S26B) and P3 (fig. S26C), respectively. Malic acid formation occurs through both P2 and P3, by the reaction of an alkoxy radical with hydroperoxyl radical (HO_2_), suggesting that the HO_2_ radical can be formed at the air-water interface, in agreement with previous observations from our group (*12*, *13*). The quantum chemistry calculations on the CA reactions show that the processes leading to product formation are all exoergic, in line with observations. The calculated energies of the species involved in the CA and MA reactions are given in tables S10 and S11.

### Evaluation of the role of O_2_

**Fig. 4.**
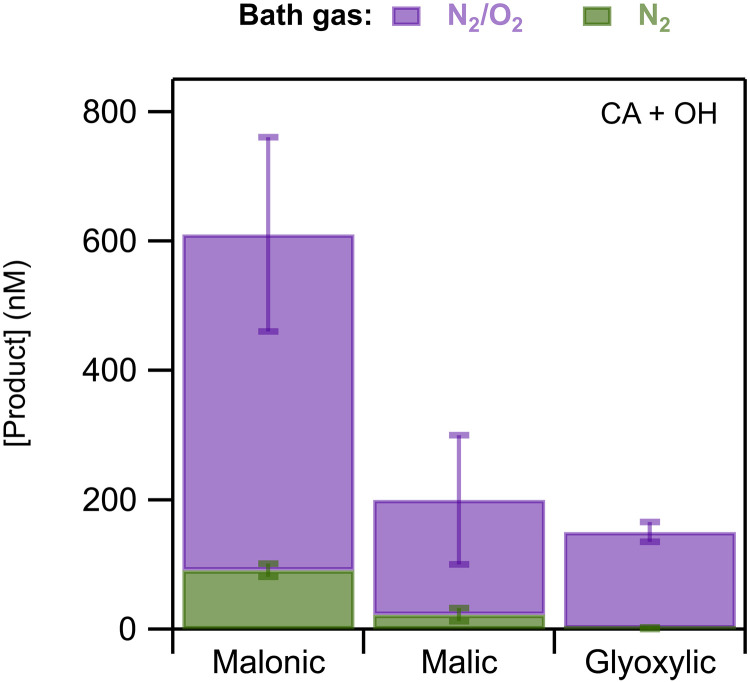
Evaluation of the role of O_2_. Comparison plot of the product concentrations in the presence (purple) and absence (green) of O_2_, produced in CA particles.

The mechanisms presented above imply the importance of O_2_ to product formation, as O_2_ initiates chain reactions that lead to the formation of stable oxidation products. O_2_ has also a major role in the spontaneous production of OH radicals at the interface, enhancing it notably, as discussed in earlier works (*12*, *13*, *16*). To confirm the important role of O_2_, we performed experiments at RH = 55% in a bath gas of nitrogen. A comparison plot of the product concentrations formed in CA particles in the presence and the absence of O_2_ is depicted in Fig. 4 (see fig. S29 for MA and AA). The particle size and mass were not affected by the change of the bath gas, and, therefore, only the absolute concentrations are given here. Overall, all the product concentrations decreased in the absence of O_2_. Specifically for CA, in an N_2_ environment malonic and malic acids concentrations decrease by factors of 7 and 10, respectively; glyoxylic acid production was negligible. Our observations confirm the suggested mechanism and the formation schemes of these products that all require the presence of O_2_. We note that O_2_ likely plays a complex role in the oxidation mechanisms, as the CA product results indicate solely the suppression of the chain reactions initiated by O_2_. However, as detailed in the Supplementary Materials, although the MA oxidation process does not involve reactions with O_2_, malic acid, the main oxidation product, decreases in an N_2_ environment (fig. S29). This finding suggests that the production of OH is greatly reduced in an O_2_-free environment, but it is not fully suppressed, in agreement with previous observations (*13*, *16*). Although the results are consistent with the literature, the fact that N_2_ experiments still led to the formation of products may also indicate that our system is not completely free of O_2_, most probably due to remaining dissolved O_2_ in the bulk solution after the bubbling process. The existence of small leaks in the experimental setup that could lead to the presence of O_2_ cannot be excluded. High–molecular weight products from radical recombination were not observed under any conditions.

## DISCUSSION

Previous studies provided evidence that OH radicals, which are spontaneously produced near the air-water interface of aqueous droplets, can initiate oxidation of organic compounds (*12*, *21*) or reactions via which H_2_O_2_ can be produced (*10*, *13*, *14*, *16*, *17*, *45*, *46*). Our experimental evidence supports these findings and offers insights into the fate of OAs, as just by adsorbing water vapor, the OA composition can change via OH-driven oxidation, which occurs at the particle surfaces and is only important in the presence of O_2_. Humidity plays a central role in particle growth (*28*), as well as in particle-phase chemical processes. Davies and Wilson (*26*) have reported enhancement of the reactive uptake of OH radicals onto CA particles under high-humidity conditions due to the lower viscosity of CA particles under these conditions. Li and Knopf (*8*) also showed that solid OAs display reactivity that increases as the particles gradually deliquesce. Therefore, the increase in product formation at the higher humidity levels observed here may also, in part, be attributed to a decrease in the viscosity of the particles, which would allow more facile diffusion of reagents. The amount of OH that is produced at each humidity level remains unknown.

The uptake of gaseous OH radicals by OA particles is a key to their tropospheric oxidation. Recently, Rasool and coworkers (*47*) predicted the heterogeneous oxidation timescale of ambient biomass burning OA for different emission scenarios: (i) 0 to 2 km, [OH]g = 3 × 10^5^ molecules cm^−3^; (ii) 0 to 2 km, [OH]g = 3 × 10^6^ molecules cm^−3^ (polluted areas); and (iii) 3 to 5 km, [OH]g = 3 × 10^6^ molecules cm^−3^. For altitudes of 3 to 5 km, the heterogeneous uptake rate constant (*k*_uptake_) varies between 2 × 10^−13^ and 6 × 10^−13^ cm^3^ molecules^−1^ s^−1^ depending on the humidity levels. Their computations showed that near the Earth’s surface (0 to 2 km) and at high OH concentration, organic oxidation occurs in less than a week, while lower backgrounds of OH increase the timescale to months. At higher altitudes, the oxidation can range between a few weeks up to months, depending on the RH of the troposphere, which defines the *k*_uptake_ and thus the decay process.

**Fig. 5.**
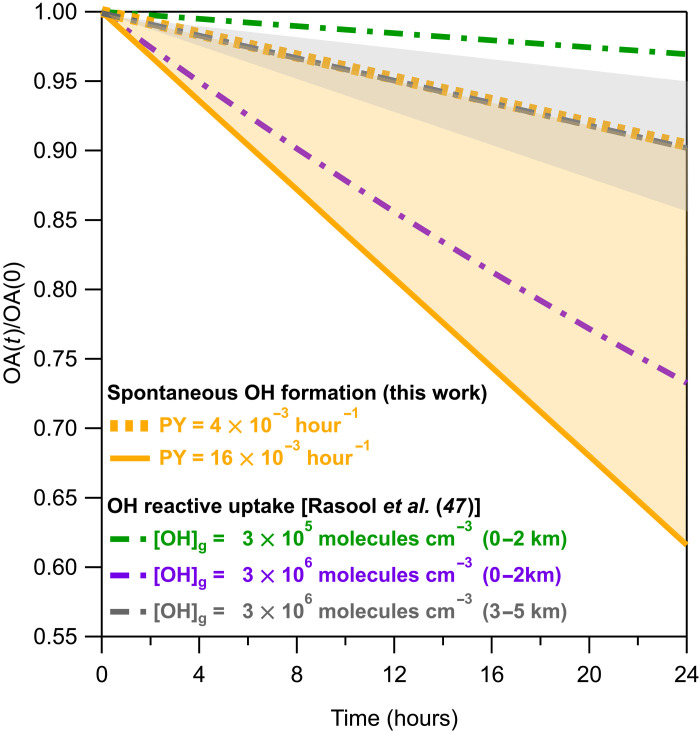
Atmospheric impact. OA decay rates due to (i) the spontaneous OH oxidation for the range of product yield of 4 × 10^−3^ to 16 × 10^−3^ hour^−1^ (orange region), (ii) the heterogeneous oxidation by OH uptake at [OH]_g_ = 3 × 10^5^ to 3 × 10^6^ molecules cm^−3^, at altitudes of 0 to 2 km (green and purple), and (iii) the heterogeneous oxidation by OH uptake at [OH]_g_ = 3 × 10^6^ molecules cm^−3^, at altitudes of 3 to 5 km and *k*_uptake_ of 2 × 10^−13^ to 6 × 10^−13^ cm^3^ molecules^−1^ s^−1^ (gray region; gray solid line is the mean value). Data for OH reactive uptake were reproduced from the study of Rasool *et al.* (*47*).

Figure 5 displays the box model predictions of Rasool *et al.* (*47*) for these three different scenarios within a 24-hour period, including only OH uptake initiated reactions. To assess the atmospheric importance of the findings described above, we compare the oxidation of OA via the spontaneous formation of OH with the one initiated by OH uptake, within the timescale of 24 hours. Here, we assume that the yields obtained in the monodisperse particles are similar to those of the polydisperse. In the flow tube reaction time of 90 s, the product formation yields of CA were measured to be 1 × 10^−4^ to 4 × 10^−4^, which corresponds to 4 × 10^−3^ to 16 × 10^−3^ per hour. The CA decay [CA(*t*)/CA(0)] was calculated on the basis of the measured product yields by assuming that the reaction of OH radicals with CA is first order, as the concentration of the organic is much higher than that of OH (text S7). The same calculations were also performed for MA, which undergoes much more rapid reactions (*38*) with higher product yields (fig. S30). Here, we emphasize the slower decay results derived from CA to compare with those of Rasool *et al.* (*47*).

The results are also displayed in Fig. 5, where the dashed yellow line shows the calculated CA loss, taking the lowest value of the measured product yield (4 × 10^−3^). The decay of the organic is estimated to be 10% within a day, which is more than 50% greater than the decay that occurs because of the OH uptake near Earth’s surface in environments of low OH concentration. We conclude that, under conditions of low ambient OH, OA decay due to the uptake of gas-phase OH may be less important to the oxidation process than the surface chemistry described here. In an OH-enriched environment, OA oxidation at low altitudes will be governed by the reaction initiated by the OH uptake into the particles. However, taking a product yield of 16 × 10^−3^ hour^−1^, the spontaneous OH formation mechanism increases the decay rate of the organic to the extent that this route becomes the major oxidation pathway in all environments. Last, for higher altitudes, the spontaneous oxidation is also expected to be an important pathway.

The results suggest that in the range of gaseous OH concentration of 10^5^ to 10^6^ molecules cm^−3^, the spontaneous formation of OH at the air-water interfaces can be a contributing oxidation process of OA particle aging that is as important as the OH uptake from the gas phase. The oxidation of OA via water surface production of OH could be even more important for organics that initiate faster reactions with OH and with higher product yields, i.e., MA (fig. S30). These interfacial OH oxidation pathways should be included in global models that also include other tropospheric degradation processes, i.e., photodissociation and Fenton reaction, to provide a more accurate prediction of the importance of heterogeneous chemistry and its atmospheric implications.

## MATERIALS AND METHODS

### Experimental apparatus and process

All experiments were carried out in a Pyrex double-walled, temperature-controlled flow tube reactor (~3 liters), at *T* = 296 K. The schematic of the experimental apparatus is given in fig. S1. Aerosol particles were generated by atomizing bulk solutions containing CA, MA, and *trans*-AA, all at 5 mM concentration, using a commercial constant output atomizer (TSI 3076). A flow of 1 liter min^−1^ of air containing the aqueous aerosol flow was passed through a diffusion dryer, reducing the RH to 3 ± 1% after the dryer, as measured by a RH and *T* probe (VAISALA, HMP110) attached at the exit of the dryer. The dried aerosol particles were introduced into the flow tube via a specific inlet; from a second inlet, dry compressed air (1 liter min^−1^) was added. To humidify the additional flow, the air passed through water bubblers. To achieve RH values between 0 and 50% RH inside the reactor, we combined different ratios of dry and humidified air. In all cases, the total additional flow was 1 liter min^−1^. An RH of 75% was achieved by putting two bubblers in series, with the first one to be heated at 35°C. For 99% RH, we added water inside the reactor. The humidity inside the reactor was also measured by an RH/*T* probe attached to its outlet. The total flow of 2 liter min^−1^ (particles and air) resulted in a residence time for particles within the reactor of ~90 s. A scanning mobility particle sizer (electrostatic classifier 3080 combined with condensation particle counter 3776, TSI) was connected to the outlet, and the total aerosol number, mass, surface, and volume concentrations were recorded.

For the RH dependent experiments, we performed offline measurements for the chemical characterization and quantification of the generated particles. The particles were collected onto a filter and analyzed using ultrahigh-performance liquid chromatography (UHPLC; Dionex Ultimate 3000, Thermo Fisher Scientific) coupled with a diode array ultraviolet-visible detector and interfaced with an HRMS (Q Exactive Hybrid Quadrupole-Orbitrap mass spectrometer, Thermo Fisher Scientific), The details description of the analytical procedure is given in the Supplementary Materials (text S1 and figs. S1 and S2).

### Size-resolved measurements

To investigate the effect of the surface-to-volume ratio on the product yield formation, we performed experiments in which monodispersed particles were introduced in the flow tube. The dry polydisperse aerosol flow passed through an AAC and then entered the reactor. AAC classifies the aerosol particle size by its relaxation time, which is related to the Da. The advantage of this method is that the particles are classified without being electrically charged, as it happens when a DMA is used and the Dm is registered. Furthermore, the particle losses are negligible, as the transmission efficiency of the AAC is better; in addition, it selects all the particle population, electrically charged or not (text S4). To investigate whether potential free electrical charges that may exist in our droplets affect the spontaneous OH radical production and thus the oxidation of the organic species, measurements were carried out with the size selection performed by a DMA; in this way, the particles are charged before their entrance into the reactor.

For the size-resolved measurements, we performed both offline and online analysis. For the offline measurements, the particles were collected using a commercial aerosol collector (Series 110B Spot Sampler) and analyzed with UHPLC Q-Orbitrap mass spectrometry (text S1). For the online chemical characterization of the monodisperse particles, the outlet of the reactor was connected to the particle-phase WALL-E inlet coupled with a Vocus AIM high-resolution mass spectrometer. Details are given in text S5.

## Supplementary Materials

### This PDF file includes:

Text S1 to S7 Figs. S1 to S30 Tables S1 to S11 References
