# Supplementary material for: Humidity drives spontaneous OH oxidation of organic particles
Source: Sci Adv. 2025 Jun 20;11(25):eadx4507. doi: 10.1126/sciadv.adx4507 (PMC12180490; doi:10.1126/sciadv.adx4507)
Supplement: Supplementary file 1 — Text S1 to S7 Figs. S1 to S30 Tables S1 to S11 References [file sciadv.adx4507_sm.pdf]

Science Advances
AAAS

Supplementary Materials for

Humidity drives spontaneous OH oxidation of organic particles

Maria Angelaki *et al.*

Corresponding author: Christian George, christian.george@ircelyon.univ-lyon1.fr

Sci. Adv. **11**, eadx4507 (2025)
DOI: 10.1126/sciadv.adx4507

This PDF file includes:

Text S1 to S7
Figs. S1 to S30
Tables S1 to S11
References

Supplementary Text

Text S1: Particle chemical characterization and quantification procedure

Offline measurements were performed for the chemical characterization and quantification of the generated particles. The particles were collected onto a 47 mm quartz filter, through a particle sampler that was attached at the outlet of the reactor. The sampling time was 2 hours for all the experiments. The filter was doped with camphorsulfonic acid (CSA, $C_{10}H_{16}O_4S$, Sigma Aldrich 99%) solution of 48μM concentration that enables the calculation of the extraction efficiency (f_{eff}) and therefore the accurate quantification of the compound concentrations, according to Eq S1.

$$[Organic] = \frac{[Organic]_{measured}}{f_{eff}} \quad \text{[Eq S1]}$$

The filter was cut into small pieces and placed into a brown vial to avoid light exposure. The vial was filled with 6 ml of acetonitrile (ACN). The mixture was agitated for 30 min, using an orbital shaker at 1000 rpm. Then it was filtered with a syringe connected to a PTFE filter, to remove insoluble matter from the filter and transferred in a second vial. The procedure was repeated to ensure high extraction efficiency, which varied between 87 % and 95 %. Finally, around ~12 ml of ACN solution that contains the filter extract was fully evaporated, using a gentle flow of pure N_2. The residues were reconstituted in 1 ml of water. The analytes were analyzed using ultra-high performance liquid chromatography (UHPLC, Dionex Ultimate 3000, Thermo Scientific) coupled with a diode array UV-Vis detector and interfaced with a high-resolution mass spectrometry (HRMS, Q-Exactive Hybrid Quadrupole-ESI-Orbitrap mass spectrometer, Thermo Scientific), following the procedure described in the study of Wang et al.(*48*). The mass resolving power of the instrument is 140,000 at m/Q = 200 Th. For the accurate characterization of the detected peaks, external mass calibration was performed on a daily basis using a 2 mM sodium acetate solution, which provides multiple negative and positive adduct ions in the range of m/Q 50–750 Th. After the mass calibration, the error was below 0.5 ppm. The data were processed using Xcalibur 2.2 software (Thermo Scientific).

The products detected in CA, MA, and AA particles are given in Tables S1 – S3. To quantify the organic species detected (both reactants and products), commercial compounds were purchased, and calibration curves were constructed. Fig. S2 shows the integration area of the citric acid, maleic acid, aconitic acid, malonic acid, malic acid, and glyoxylic acid peaks, as a function of the solution concentration. The calibration factors (C_f) for each compound were calculated via equation Eq S2 and they are given below:

$$[Organic] = \frac{Integration\ area}{C_f} \quad \text{[Eq S2]}$$

Citric acid: $C_f = (16.8 \pm 0.30) \times 10^6$, maleic acid: $C_f = (24.9 \pm 0.40) \times 10^6$, aconitic acid: $C_f = (4.13 \pm 0.08) \times 10^6$, malonic acid: $C_f = (2.34 \pm 0.04) \times 10^6$, malic acid: $C_f = (1.35 \pm 0.07) \times 10^7$ and glyoxylic acid: $C_f = (6.00 \pm 0.09) \times 10^6$.

Chemicals

The chemical compounds were all purchased from Sigma-Aldrich: citric acid ($C_6H_8O_7$, ≥99.5%), maleic acid ($C_4H_4O_4$, ≥99%), trans-aconitic acid ($C_6H_6O_6$, 98%), glyoxylic acid ($C_2H_2O_3$, 50% wt solution), malonic acid ($C_3H_4O_4$, ≥99%) and DL-malic acid ($C_4H_6O_5$, ≥99%). All the solutions were prepared using ultrapure water (Elga Purelab Classic, 18.2 MΩ). The air was produced via a compressed air generation system that was equipped with air cooler, dryers, and filters, ensuring the removal of impurities i.e., water, oil, and particles.

Text S2: Formation of OH oxidation products in MA and AA particles at different humidity levels

In the main text we discuss the products formation in CA particles as a function of RH. The chemical structure of the products is given in Fig. S3. In Fig. S4 we give the products concentration normalized by the total particle surface at different humidity levels. The normalized concentrations display the same trend as that of the absolute and normalized by the mass concentrations that are presented in Fig. 1.

In MA particles, malic acid was produced under humidified conditions. Humidified AA particles led to the formation of malic and glyoxylic acids. To ensure that the formation of the products occurs exclusively in the particle phase, the bulk solutions used for the nebulization were also analyzed before and at the end of the experiment (duration is 2 h), using LC-HRMS; none of these products were observed in the bulk solutions. To avoid oxidation of the samples that may occur in several days due to the air-water interface of the solution, for each experiment a fresh solution was prepared. The product concentrations increase as a function of RH, in all cases (Fig. S5 – S6), as discussed in the main text. Unlike CA and MA, AA has an absorption band in the UV-Vis region (up to 460 nm), suggesting that photochemistry can occur.(*49*, *50*) All experiments were carried out under dark conditions to prevent any light-induced reactions.

In the bulk solutions of MA, acrylic acid was detected and its production was stable at different humidity levels. Acrylic acid peak overlaps with the one of MA (same retention time) suggesting

that they cannot be separated in the column of LC and/or acrylic acid may be formed in the region of HESI source of the mass spectrometer. Finally, although malonic acid was seen to increase in the particles with increasing relative humidity, it was also measured as an impurity in the bulk solutions. For this reason, we will focus mainly on the malic acid product from maleic acid.

Text S3: Particle size distribution as a function of humidity

Fig. S8 – S10 show the total number (upper panel), mass (middle panel) and surface (bottom panel) particle size distribution data as a function of the electrical mobility diameter (Dm), for all the organic compounds, at different levels of RH. The size and the total mass and surface concentration of CA, MA and AA particles display a systematic increase as a function of humidity, suggesting that water is adsorbed on the particles, resulting in their hygroscopic growth. No notable growth was observed at 99 % RH, which is contradictory with the hygroscopic growth of the particles that is reported in literature. We attribute this phenomenon to the high sheath flow of the DMA column that shrinks particle size when their water content is high. (*51*) The mean diameter and total particle mass for each experiment are given in Tables S4-S6.

Text S4: Offline size-resolved measurements

First, we measured the correlation between the electrical mobility (Dm) and aerodynamic (Da) diameter by recording the size distributions with the two instruments, under the same conditions (Eq S3).

$$D_a = D_m\sqrt{\rho_p} \qquad \text{[Eq S3]}$$

Fig. S11 shows the particle concentration as a function of Dm and Da, for all the carboxylic acids. In all cases, the total concentration of particles is higher when the AAC is used compared to the DMA, for reasons mentioned above. For CA, the maximum Dm and Da are 105.5 and 158.8 nm respectively, leading to an average particle density of ρ_p = 1.90 g cm^{-3} (CA density is 1.7 g cm^{-3}). For AA, the ρ_p found to be 1.87 g cm^{-3}, with Dm and Da of 113.4 and 155.6 nm, respectively. These derived densities of CA and AA particles are slightly higher than those of the corresponding acids. For the case of MA, the particle density was calculated to be ρ_p = 2.40 g cm^{-3} (Dm = 117.6 nm and Da = 278.9 nm), a value much higher than the density of maleic acid (1.54 g cm^{-3}). The reasons for these deviations remain unclear. However, we suspect that this is may be due to the total charged particle population being higher, leading to an increase of the size and number of

particles when the AAC is used. Although this is a possible explanation other reasons cannot be excluded. For example, when wet particles pass through the DMA evaporation processes may occur due to the high sheath flow (5 L min^{-1}) that the instrument uses, leading to the decrease of the particle size. This phenomenon is expected to be more intense inside the DMA column as AAC sheath flow is five times lower.

As mentioned in the main text, at the outlet of the reactor, the monodisperse particles were collected using a commercial aerosol collector (Series 110B Spot Sampler^{TM}) and analyzed offline. The collector uses a three-stage condensational system to enlarge aerosol particles, by exposing them to supersaturated water environment. The particles that now contain water are directed through a nozzle into a small vial. The volume of the collected liquid particles was ~ 200 μL. The collected liquid was doped with CSA, ensuring the accurate quantification. Eq S1 was used; in this case f_{eff} is the correction factor of dilution. The liquid samples were analyzed with UHPLC/Orbitap-MS, as described in Text S2. For the quantification of the product yield, the concentration of each product was divided by the concentration of the reactant according to Eq S4.

$$\% \, PY = \frac{[Product]}{[Reactant]} \times 100 \qquad \text{[EqS 4]}$$

The particle size distribution was also measured after the reactor, using an SMPS. Fig. S12 – S14 depict the monodisperse electrical mobility size distributions (concentration at the upper panel and mass at the bottom one) of CA, MA and AA particles, at 55 % RH. Although the selection of the particle size occurred based on particle Da, we give as an inset the Dm, which was calculated by the measured densities. In this way we can correlate same quantities and depict better the particle growth, as the recorded size distributions after the reactor are based on the electrical mobility diameter. The values of the selected Dm and Da are given Tables S7 – S9. The maximum population of the particles is located at higher diameters than the selected one, denoting particle growth. In Fig. S15 we present the malic acid formation yield as a function of the surface-to-volume ratio, produced in MA and AA particles. Glyoxylic acid was not observed as a product in AA particles due to the low total particle mass. Acrylic acid was again observed in MA particles. Its formation yield remained stable at different sizes, suggesting that its formation is due to an artifact of the LC-HRMS, as mentioned above. Similar to CA, the reaction of OH with MA and AA is favored in smaller droplets, leading to the increase of the product yields.

Finally, in Fig. S16, we display the size distributions (recorded after the flow tube), when CA particles of 108.1 nm of diameter were selected with AAC and DMA. The DMA results are consistent with those obtained with the AAC, regarding the growth of the particles; the population has a maximum peak at 129 nm in both cases. However, shoulders appeared that correspond to double charged particles. A notable difference is the decrease of the particle concentration and mass, attributed to the higher transmission efeciency of the AAC, as above-mentioned. As described in the main text, the presence of charges did not affect the formation yields of the products.

Text S5: Online size resolved measurements

For the online chemical characterization of the monodisperse particles, the outlet of the reactor was connected to the WALL-E (wall free particle evaporator) particle phase inlet coupled with a Vocus AIM (adduct ionization mechanism) high resolution mass spectrometer. The technical characteristics of the instrument are presented elsewhere and only a short description is given here.(*42*, *43*) The WALL-E inlet consists of (a) a charcoal denuder, which ensures the removal of all the gas phase components; (b) a heated inlet via which N_2 is supplied; (c) a second heated inlet (TD), located at the same place as the first one, enabling the evaporation of the particles and prevents contact between the sample and walls; and (d) an inlet via which N_2, in ambient temperature, is supplied, achieving dilution and cooling of the gas flow. In the end, gas phase components enter the Vocus AIM. The reagent ions are generated after the entrance of the Vocus reactor. For the experiments of this study, iodide (I^-) was selected as the reagent ion, as it is suitable for the detection of carboxylic acids. I^- was generated by heating a permeation tube (T = 80 ºC) containing a mixture of benzene (C_6H_6, Sigma Aldrich, ≥ 99.9 %) and methyl iodide (CH_3I, Sigma Aldrich, 99.8 %). The gaseous mixture was transported, via N_2, into a vacuum ultraviolet (VUV) lamp housing. During the exposure of the mixture with the VUV lamp, benzene absorbs the light and generates electrons, which react with the CH_3I, producing I^- according to the following scheme (RS1 – RS3)

$$C_6H_6 + hv \rightarrow C_6H_6^+ + e^- \quad \text{[RS1]}$$

$$CH_3I + e^- \rightarrow CH_3 + I^- \quad \text{[RS2]}$$

$$X + I^{-} \rightarrow XI^{-} \quad \text{[RS3]}$$

The reagent ions react with the analytes forming the iodide adducts that are finally detected via time-of-flight mass spectrometry. The instrument measures a mass-to-charge (m/Q) range of 1–900 Th with a mass resolving power of 10,000–11,000. The molecular formula and the exact masses of the detected organic species are given in Tables S1 – S3.

WALL-E parameters

The high temperature of the TD ensures a flash particle evaporation. However, high temperatures may initiate secondary processes, such as thermal decomposition of the compounds and/or their dehydration as observed in other techniques (*52*). To achieve maximum evaporation and minimal fragmentation, we performed experiments where the TD was heated from 100 to 400 °C while maintaining a constant hot sheath flow. The signals of CA, MA and AA were recorded, as well as those of the major fragments that were observed. The results are displayed in Fig. S17 – S19. Citric acid signal increases as a function of temperature; and reach its maximal desorption at 225 °C, after that a slight decrease of the CA signal is observed indicating minimal fragmentation (i.e., < 6 %). In agreement with Yang et al., (*7*) the fragment is $C_6H_6O_6$, which is formed by the dehydration of CA. Then, $C_6H_6O_6$ undergoes dehydration and decarboxylation forming $C_6H_4O_5$ and $C_5H_6O_4$, respectively. Maleic acid signal displays increase with a maximum at 150 °C and negligible contribution of fragments. At higher temperature, MA is subjected into dehydration, forming maleic anhydride. The highest signal intensity for the AA was recorded at 175 °C, with the contribution of its fragments, $C_6H_4O_5$ and $C_5H_6O_4$, to be 11 and 3 % respectively. Considering all the above results TD temperature was set at 225 °C, 150 °C and 175 °C for the CA, MA and AA measurements, respectively.

Product yields – Online measurements

For the determination of the product yields, different sizes were selected and the signals of reactants and products were monitored. The time series of the signals are displayed in Fig. S20 – S22. The product yields as a function of the surface-to-volume ratio are given in Fig. S23 – S25. The calculation of product yield was carried out by dividing the signal of each product by the

signal of the reactant. This qualitative comparison considers comparable sensitivity for the multi-functionalized compounds observed within this work.

In agreement with the offline measurements, malonic acid was generated in CA particles and malic acid in CA, MA, and AA particles. All these products increase linearly with the surface-to-volume ratio. Glyoxylic acid was also observed (CA and AA). However, the signals were unstable, and thus the quantification was not possible with the Vocus AIM, most probably due to the higher volatility of this compound.

Due to the high sensitivity that online measurements provide, additional products were observed in all cases. Acrylic acid and $C_4H_4O_5$ are produced during the OH oxidation of MA and AA, respectively. $C_4H_4O_5$ may correspond to oxalacetic acid and/or hydroxy maleic acid and its formation is promoted in particles of higher surface-to-volume ratio (linear or exponential increase). In contrast with the offline measurements, acrylic acid production displayed an increase in smaller particles. This suggests that acrylic acid may be produced in MA particles. It is worth mentioning that compounds that were detected due to the organic fragmentation at the WALL-E inlet i.e., aconitic acid from citric acid or maleic anhydride from maleic acid, did not display any size dependence. Therefore, acrylic acid could be an oxidation product that needs further investigation and more accurate quantification, which were beyond the scope of this work.

Text S6: Mechanistic investigation

In this section, we present proposed mechanisms schemes that yield the observed products. One aspect of these involves the reactive decarboxylation of parent species to form reactive radicals. A full exploration of this mechanism will be the subject of a future paper. Quantum chemical calculations were performed to confirm the viability of some key steps of the mechanisms proposed here. The calculations were carried out using the Spartan 24 set of programs for the Windows operating system.(*53*) For the citric acid pathways, geometries were optimized using Density Functional Theory methods at the B3LYP/6-311+G** (or higher) level, and final DFT energies were calculated at the ωB97X-V/def2-TZVPPD level. For the maleic acid pathways, geometries and transition states were optimized at the ωB97X-D/6-31+G* (or higher) level, and final energies at the ωB97X-V/def2-TZVPPD level. Transition states were confirmed by the existence of a single imaginary frequency, which described motion along the presumed reaction

co-ordinate. Tables S10-S11 report the energies used here. The reaction energetics were calculated from these higher-level results, and reported in units of kJ/mol.

The mechanism for the CA reactions is described in the main text and is illustrated in (Fig. S26a-S26c). Overall, for CA, our theoretical calculations confirm the experimental measurements. Note that the formation of hydroxy maleic acid (side carboxylic group H abstraction) is an endothermic process; this product was not detected in our experiments, in line with the calculations.

MA oxidation may proceed in two ways, presented in Fig. S27. The first is the electrophilic addition of the OH radical to the double bond. The initially formed radical may then react with HO_2 or with water to form malic acid; the latter case regenerating OH as well. The barrier for this process lies well below the initial reagent energies. Alternatively, reaction with HO_2, yielding O_2 as the second product, is expected to be energetically very favourable. In a second pathway, the reaction of MA with OH can also occur via hydrogen abstraction from the carboxylic group, followed by spontaneous decarboxylation. The radical thus formed reacts with water, resulting in acrylic acid, verifying the online measurements. All barriers along this reaction path lie at most 15 kJ mol^{-1} above reactant energies. Both malic and acrylic acid formation pathways are exothermic, with the production of malic acid predicted to be energetically much more favored (-93 vs -17 kJ mol^{-1}), consistent with its much greater presence in the products.

Reactions of AA with OH may proceed via OH addition to the double bond or via reactive decarboxylation. For this reaction, we did not perform any calculations, as the product formation pathways are similar to those discussed above. They are presented in Fig. S28. The attack of the radical to the middle carbon (marked in blue in Fig. S28) and the subsequent O_2/RO_2 reactions lead to the formation of an alcoxy radical, which dissociates into glyoxylic acid and the HOOC-CH_2-$^{\cdot}$C(OH)-COOH radical. The latter forms malic acid when it reacts with HO_2, and forms hydroxy maleic acid when it reacts with O_2. Both these products were observed in our experiments. OH addition to the side carbon (marked in purple) leads to the formation of $C_4H_4O_6$, oxalacetic, glyoxylic and oxalic acids. $C_4H_4O_6$ was observed during the online measurements. However, the quantification of this product was not possible due to the low signal intensity. Glyoxylic acid signals were unstable, as it is a high volatile organic compound and it cannot be easily detected in the particle phase. Finally, oxalacetic acid is an isomer of hydroxy maleic acid and therefore the compounds cannot be separated with the instrumentation used.

Text S7: Atmospheric importance

To assess the atmospheric importance of the spontaneous OH oxidation of the organic aerosols (OA) studied here we calculated the percentage of their decay in 24 hours. To calculate the OA(t)/OA(0), we assumed that the reaction is first order, due to the much higher concentration of the organic compounds compared to the formed OH radicals at the interface. The kinetics are described by reaction RS4 and equations Eq S5 – Eq S8.

$$Organic \xrightarrow{k} Products \qquad [RS4]$$

$$\frac{[Organic]_t}{[Organic]_0} = e^{-kt} \qquad [Eq\ S5]$$

$$[Product]_t = [Organic]_0 - [Organic]_t \qquad [Eq\ S6]$$

$$Product\ Yield = \frac{[Product]_t}{[Organic]_0} = 1 - e^{-kt} \qquad [Eq\ S7]$$

$$\frac{[Organic]_t}{[Organic]_0} = 1 - Product\ yield \qquad [Eq\ S8]$$

Citric acid decay has been described in the main text as the lower limit to the rates of OA decay we consider. Here we present the data for maleic acid as the upper limit of the decay rate, as the product yield values were higher compared to those of citric acid. The main product of the reaction of maleic acid with OH radicals is malic acid with yield values between (1.5 – 5) × 10^{-3} in 90 s reaction time. In 1 hour, the yields are 0.06 – 0.2. By applying Eq S8 we calculated the maleic acid decay that is presented in Fig. S30 with a light blue color. Maleic acid decay is significantly faster than that of citric acid due to the rapid reaction of OH radicals with the double bond. While citric acid loss is approximately 60 % within 24 hours, the same loss for maleic can occur in 2 to 8 hours, depending the product yield used for the calculations. In all scenarios of the model of Rasool et al.,(*47*) the reaction of maleic acid with OH radicals that are spontaneously formed at the interfaces is the dominant oxidation pathway compared to the one of the OH uptake.

Figures

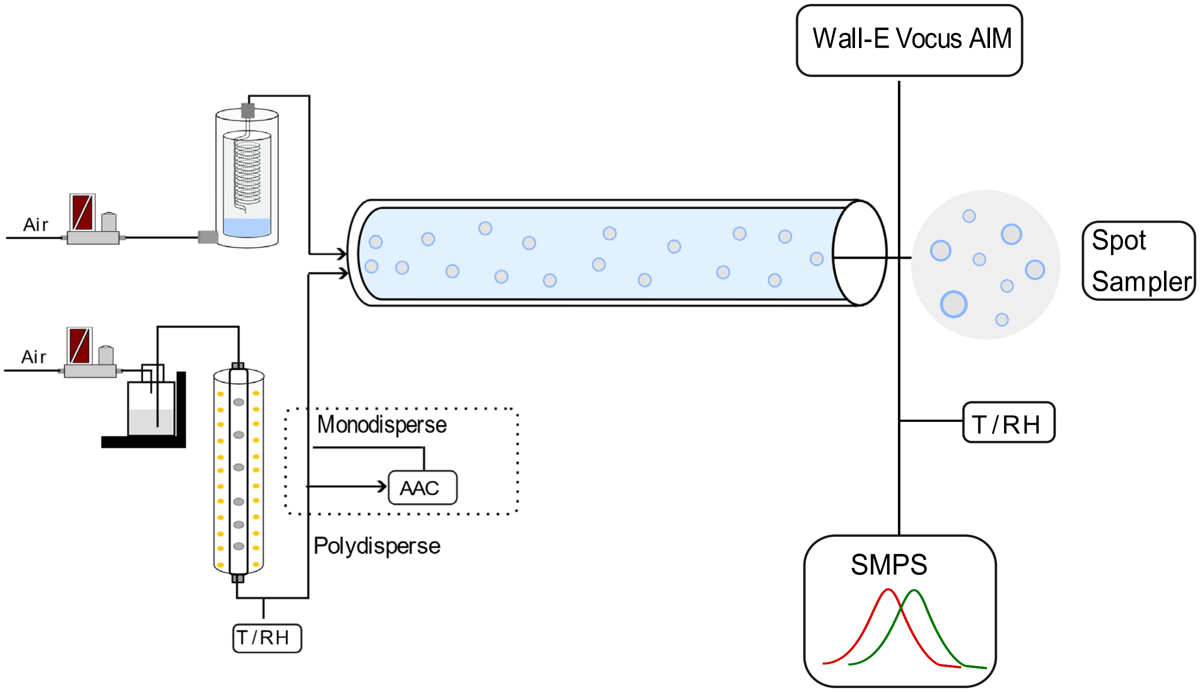

Fig. S1. Experimental apparatus.

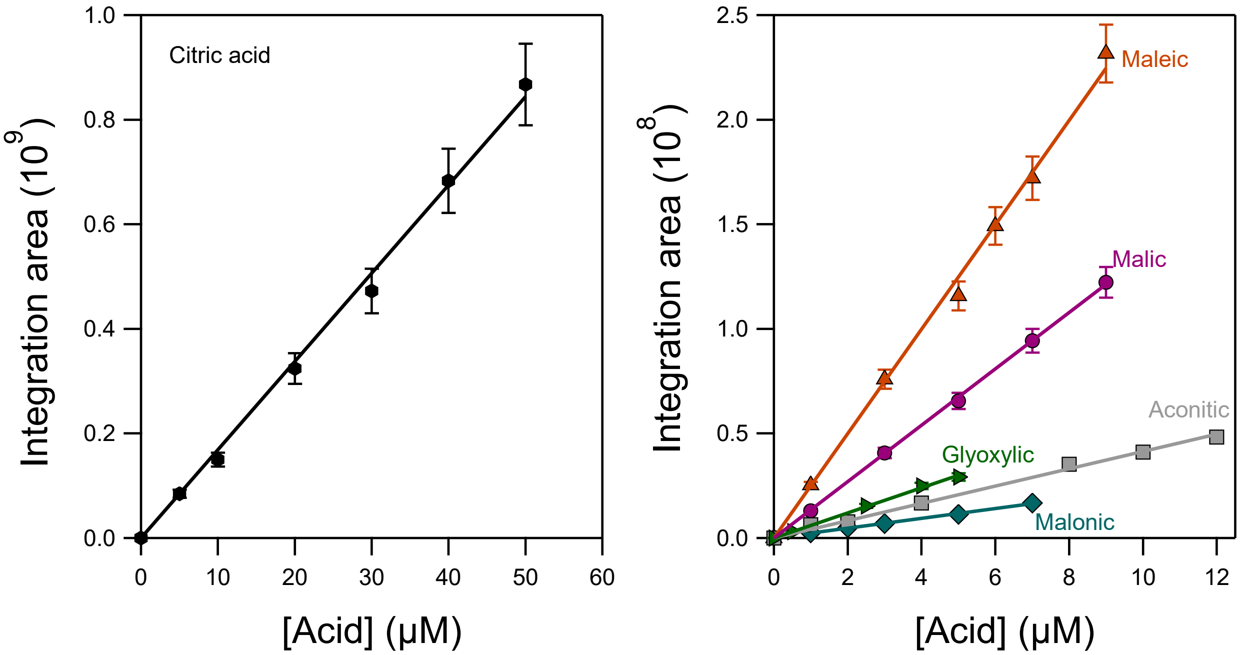

Fig. S2. Quantification of organic species using UHPLC/HRMS: Calibration curves for citric acid (black circles) maleic acid (orange triangles), malic acid (purple circles), glyoxylic acid (green triangles), aconitic acid (gray squares) and malonic acid (blue diamonds).

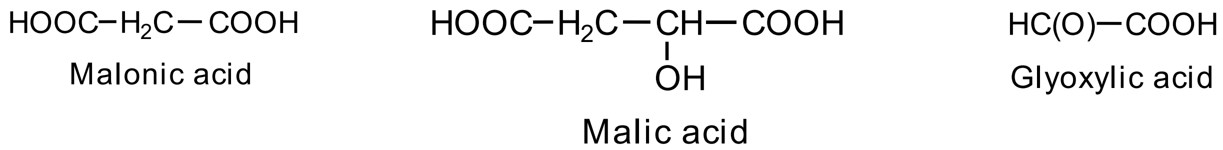

Fig. S3. Chemical structure of malonic, malic and glyoxylic acids. These are the main OH oxidation products of the studied compounds.

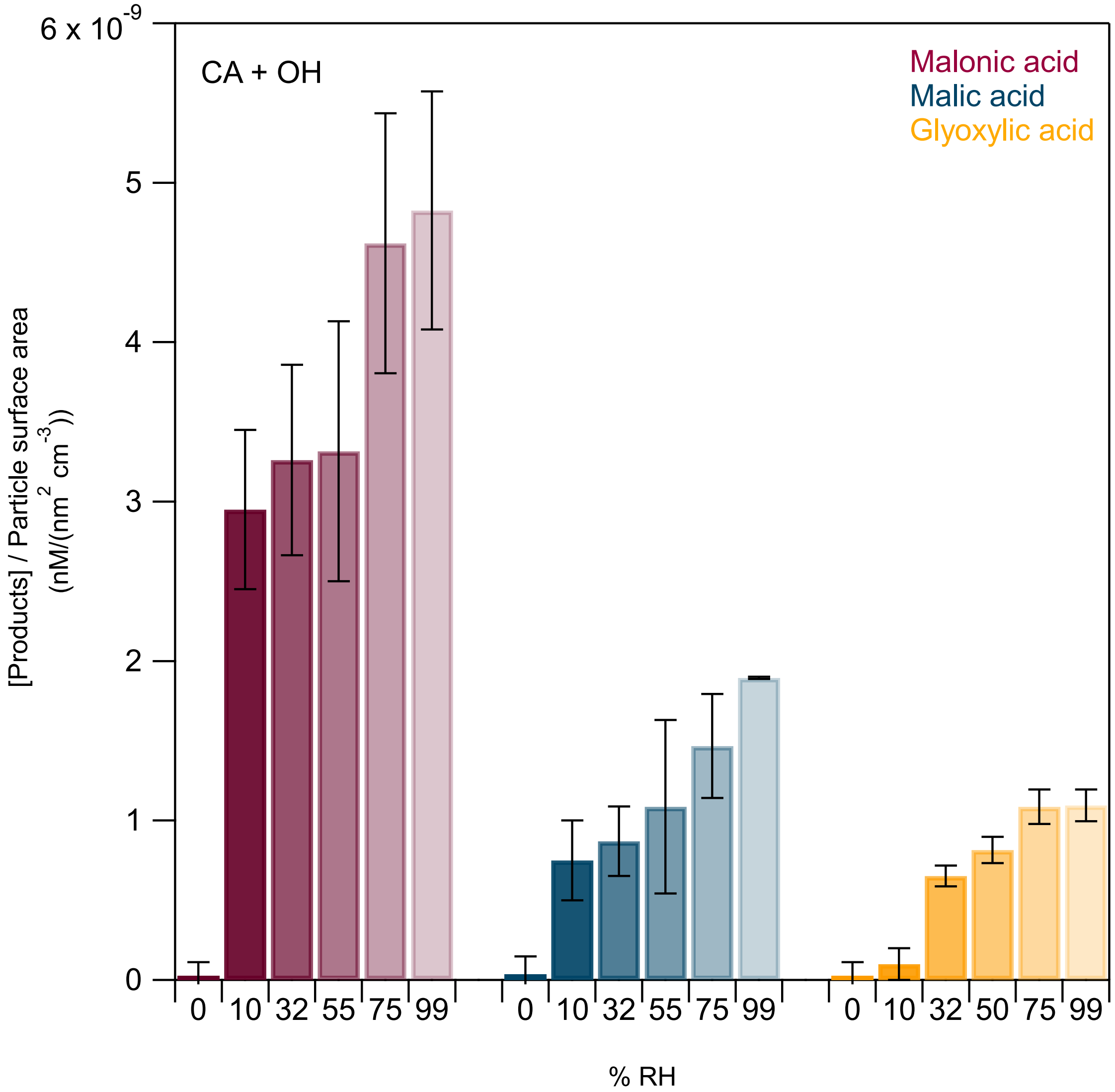

Fig. S4. Citric acid + OH: Products formation at various humidity levels. Malonic, malic and glyoxylic acid concentrations from citric acid particle oxidation as a function of RH. The RH is increasing as the color scale fades. The concentrations have been normalized by the total particle surface area.

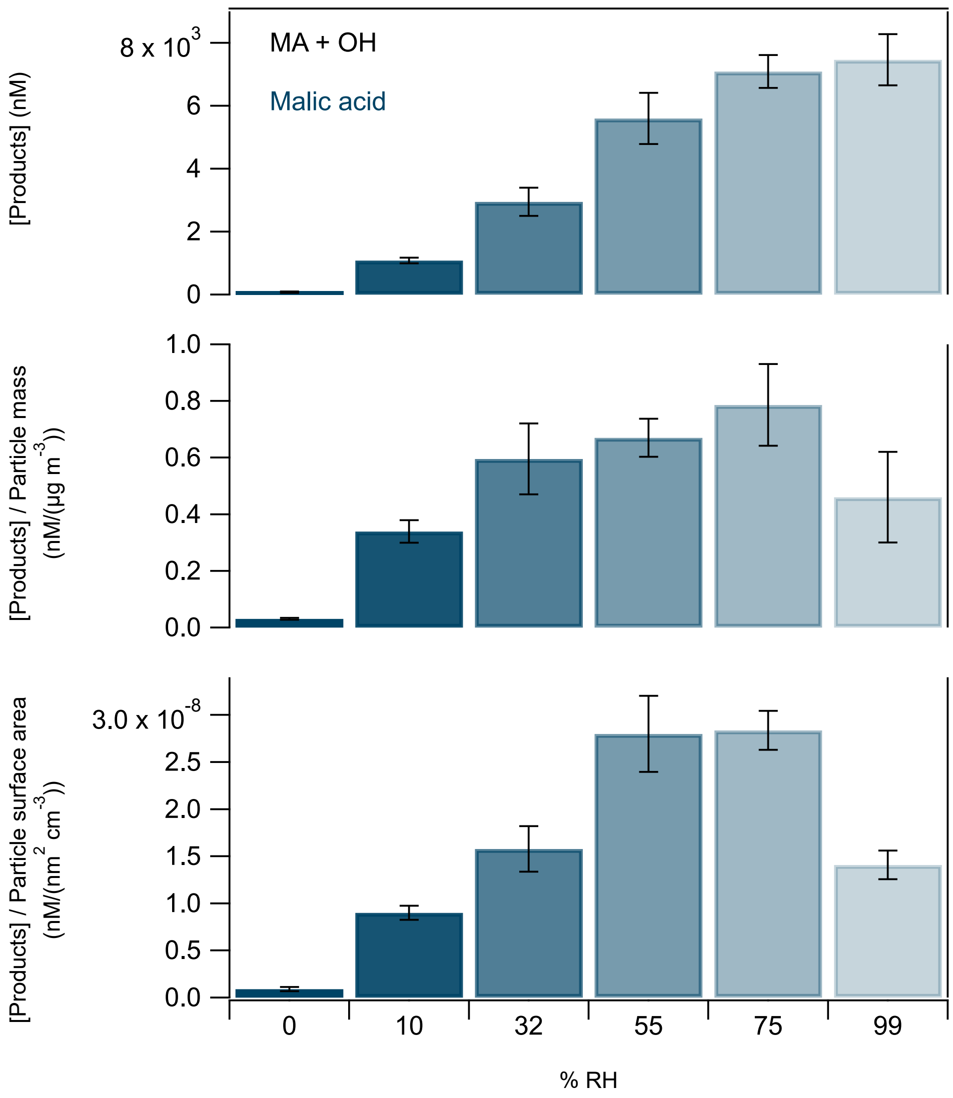

Fig. S5. Maleic acid + OH: Products formation at various humidity levels. Upper panel: malic acid concentrations from maleic acid particle oxidation as a function of RH. The RH is increasing as the color scale fades. Middle panel: malic acid concentration normalized by the total particle mass, also as a function of RH. Bottom panel: malic acid concentration normalized by the total particle surface, also as a function of RH.

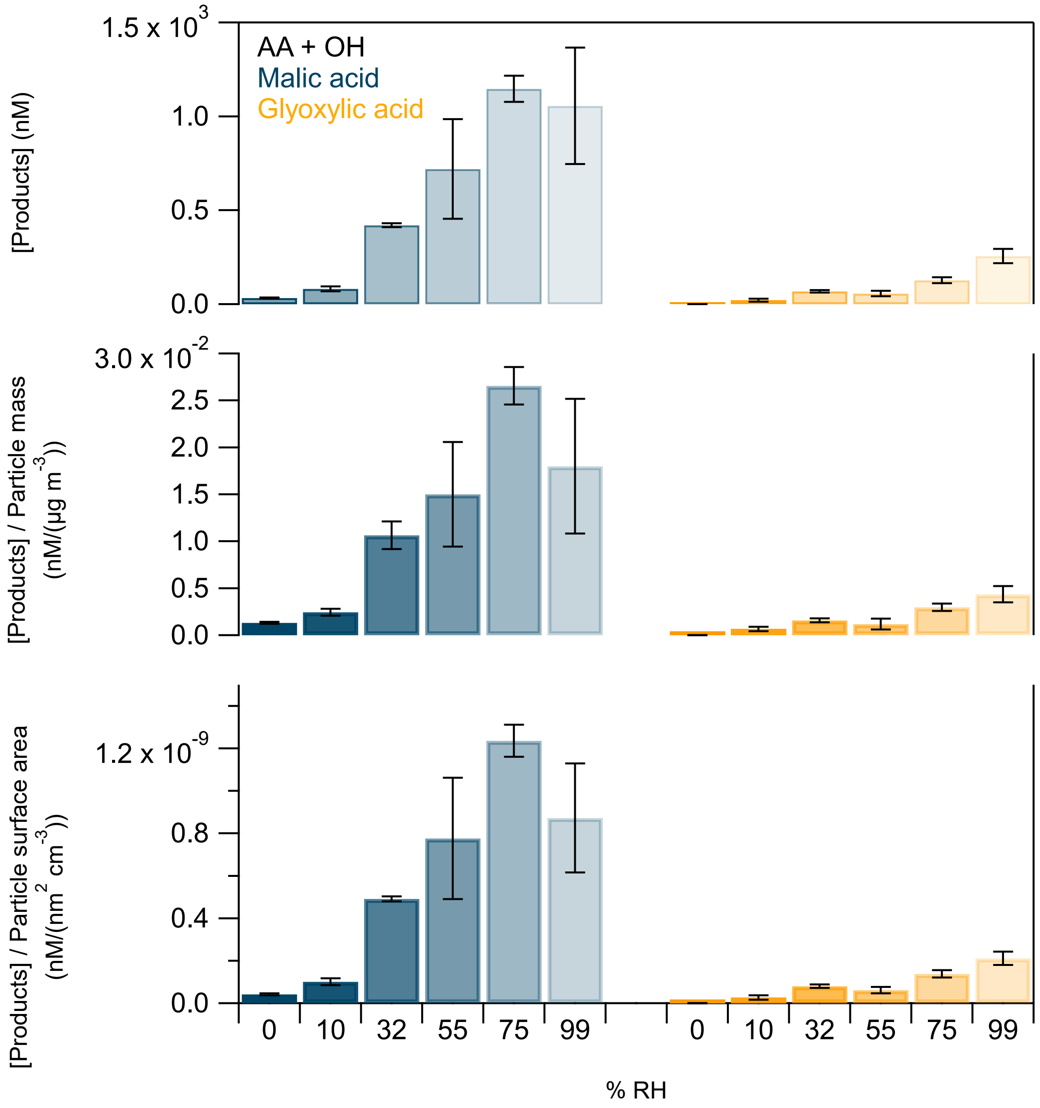

Fig. S6. Aconitic acid + OH: Products formation at various humidity levels. Upper panel: products concentration from aconitic acid particle oxidation as a function of RH. Malic acid: blue, Glyoxylic acid: yellow. The RH is increasing as the color scale fades. Middle panel: the concentration of the generated products normalized by the total particle mass, also as a function of RH. Bottom panel: the concentration of the generated products normalized by the total particle surface, also as a function of RH.

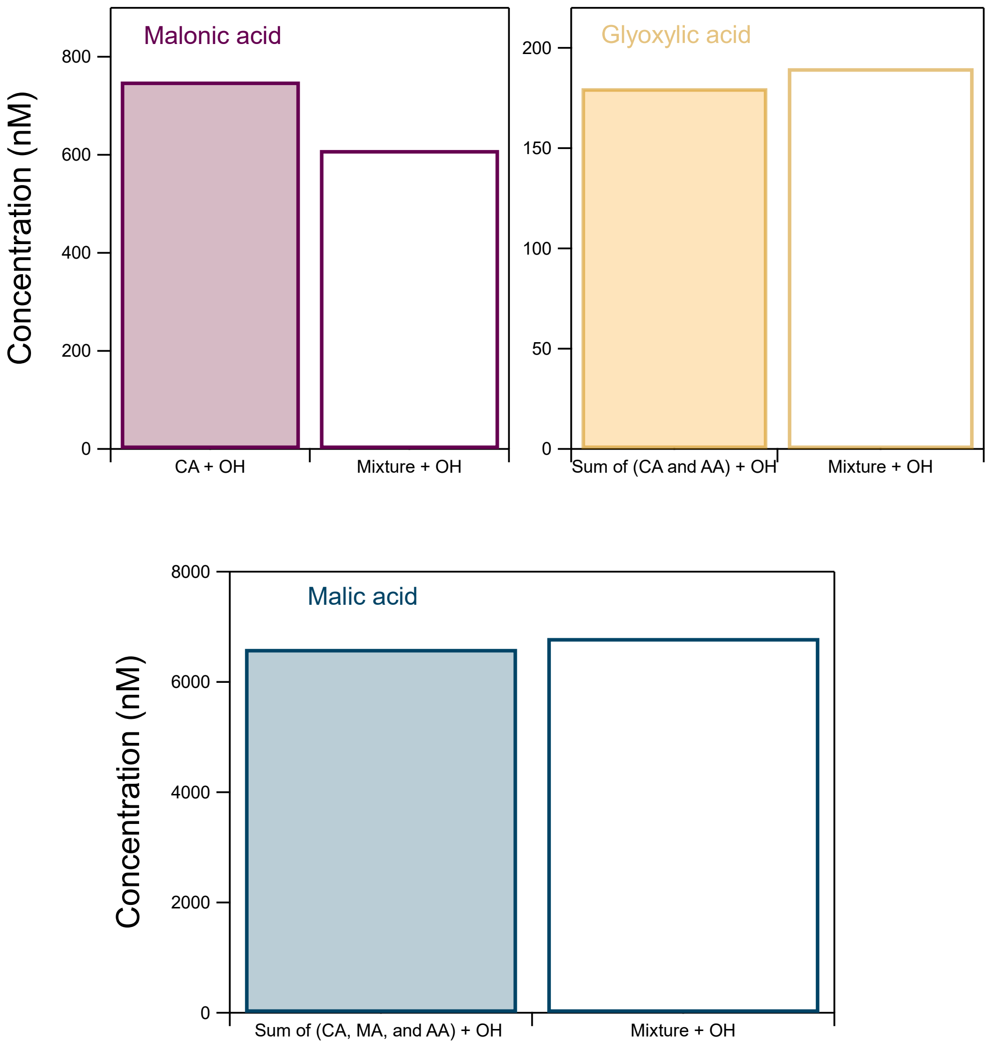

Fig. S7. Products formation in mixtures and single component particles. Malonic, malic and glyoxylic acid concentrations formed in single-component acid particles (solid bars) and mixture of citric, maleic and aconitic acid particles (open bars).

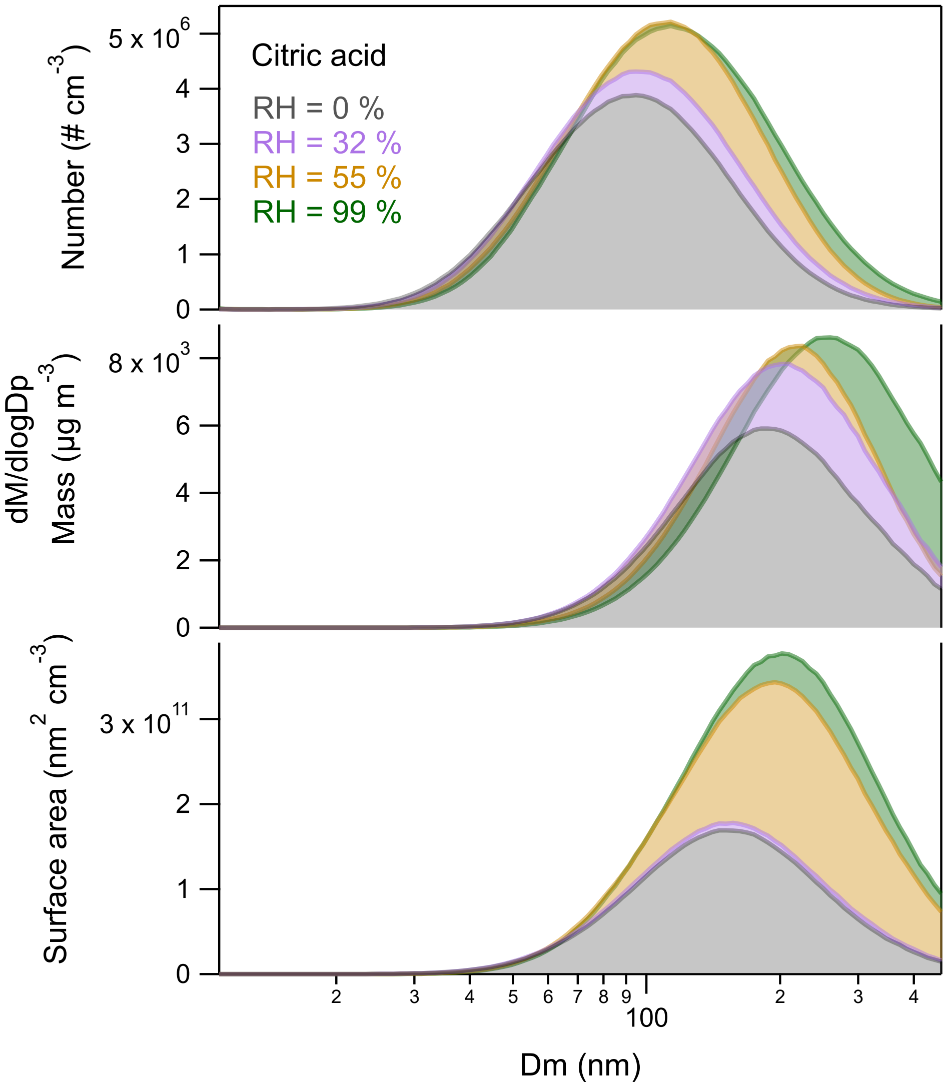

Fig. S8. Electrical mobility diameter distributions at various RH levels. Concentration (top), mass (middle) and surface (bottom) particle size distribution of citric acid. Different colors represent different humidity levels.

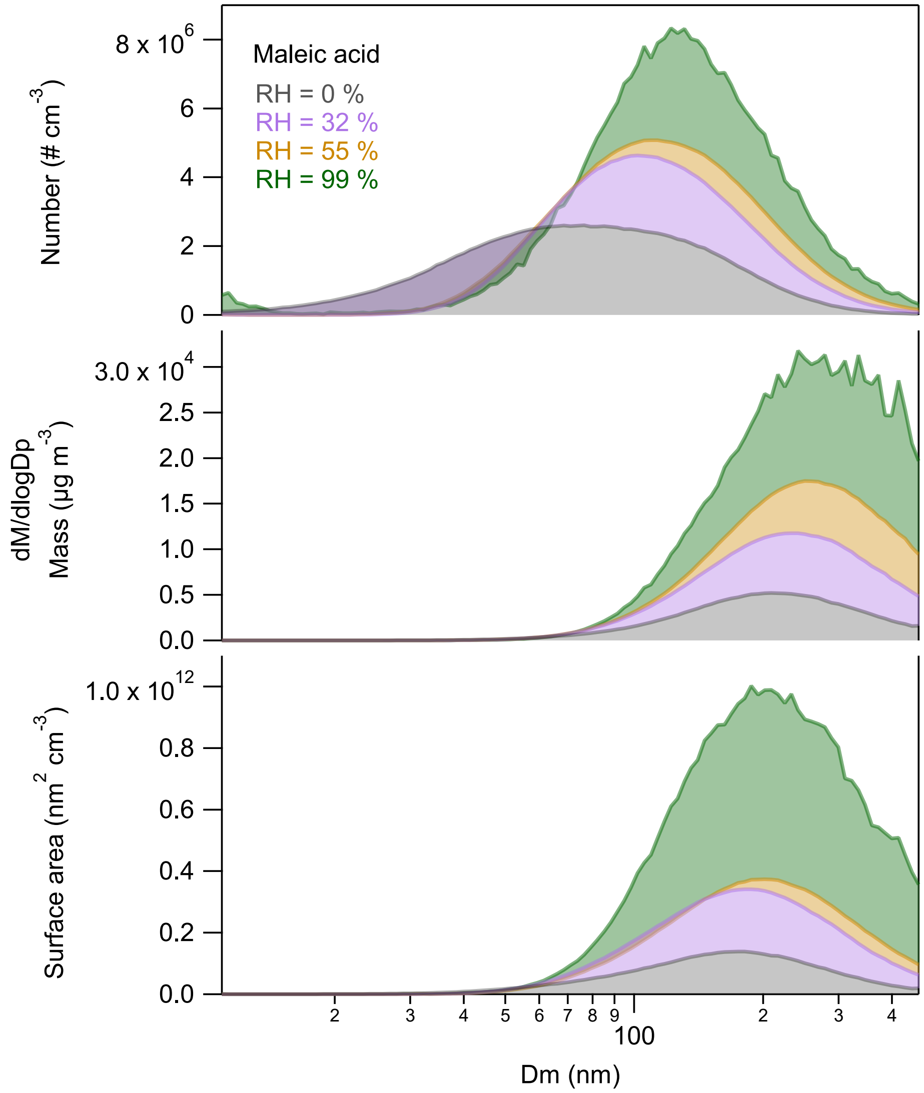

Fig. S9. Electrical mobility diameter distributions at various RH levels. Concentration (top), mass (middle) and surface (bottom) particle size distribution of maleic acid. Different colors represent different humidity levels.

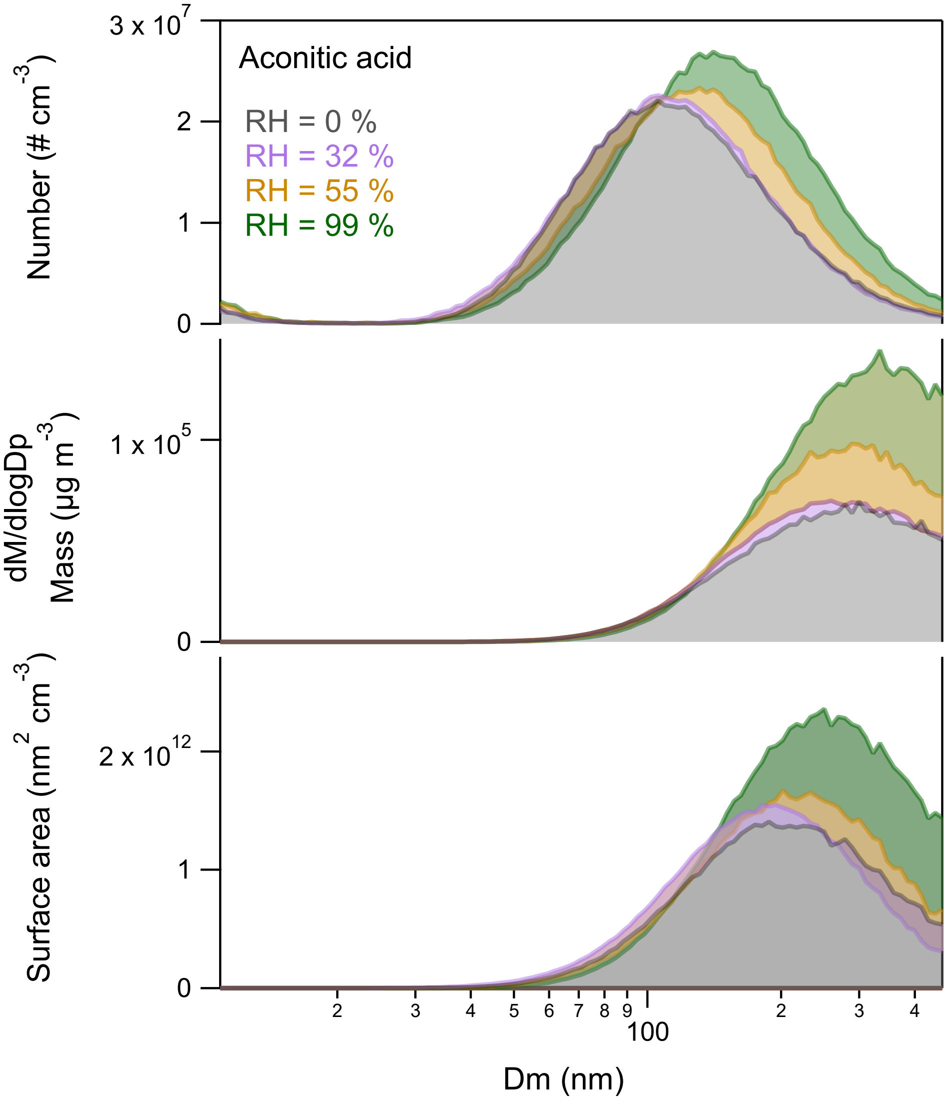

Fig. S10. Electrical mobility diameter distributions at various RH levels. Concentration (top), mass (middle) and surface (bottom) particle size distribution of aconitic acid. Different colors represent different humidity levels.

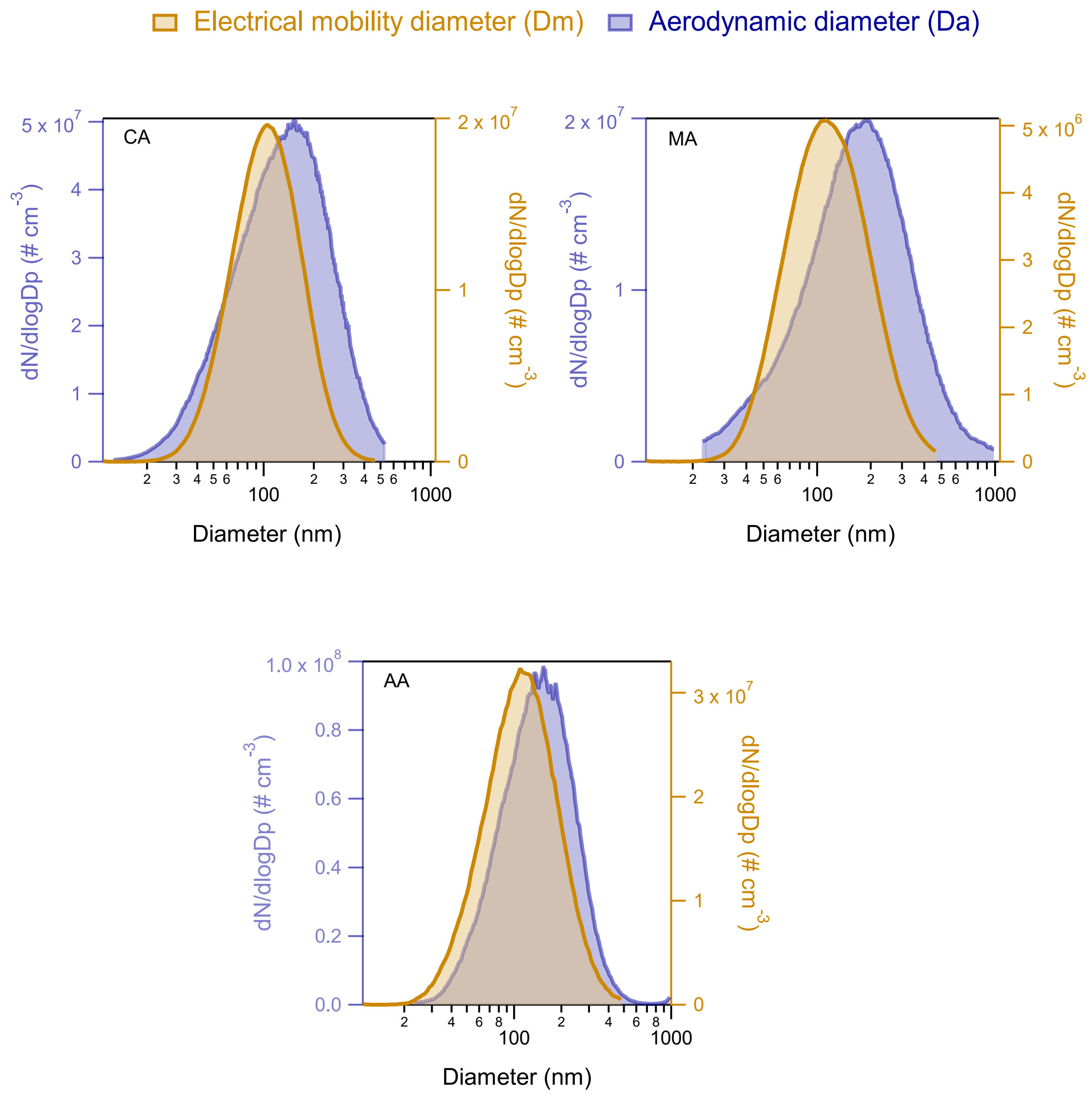

Fig. S11. Electrical mobility and aerodynamic size distributions. Dm and Da are given in brown and blue colors, respectively. Top left, top right and bottom graphs represent citric, maleic and aconitic acid particles, respectively.

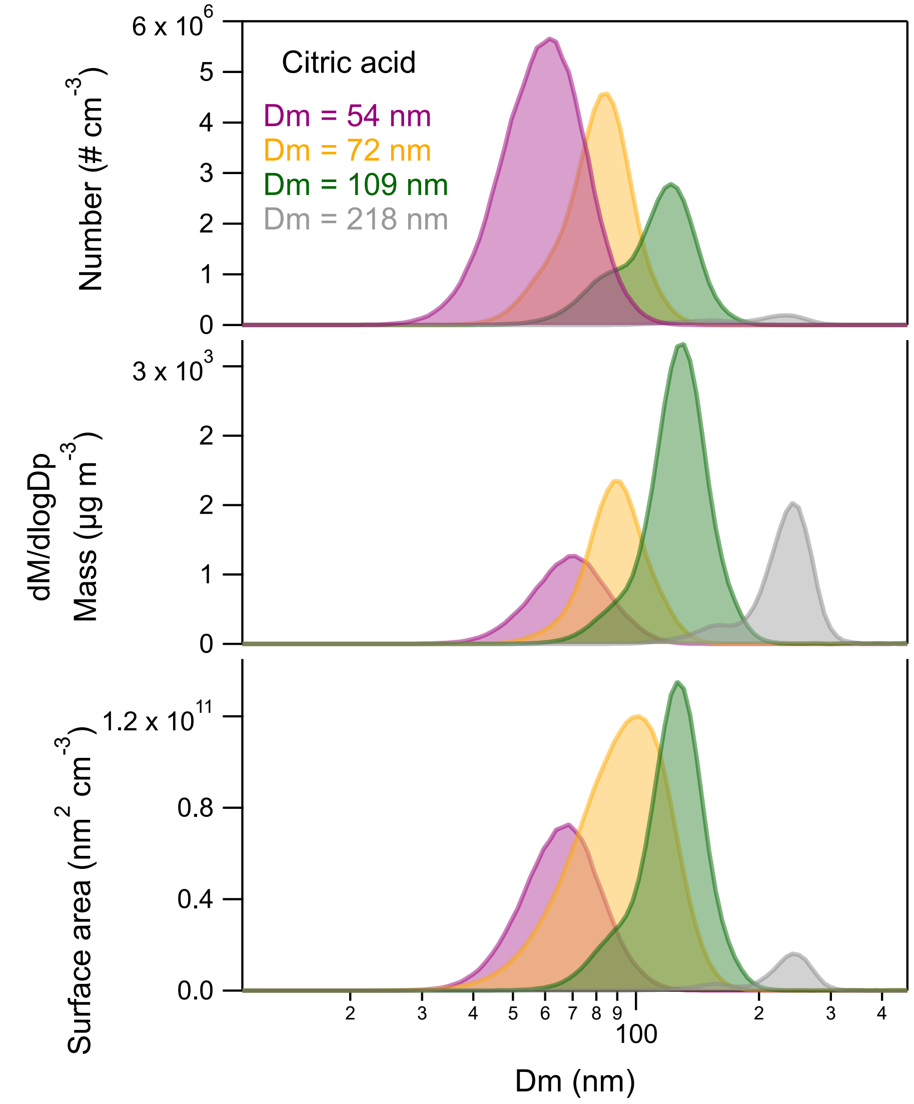

Fig. S12. Electrical mobility diameter distributions of citric acid particles (size-resolved measurements). Concentration (top), mass (middle) and surface (bottom) particle size distribution of citric acid. Different colors represent different sizes. The selected size is given as an inset.

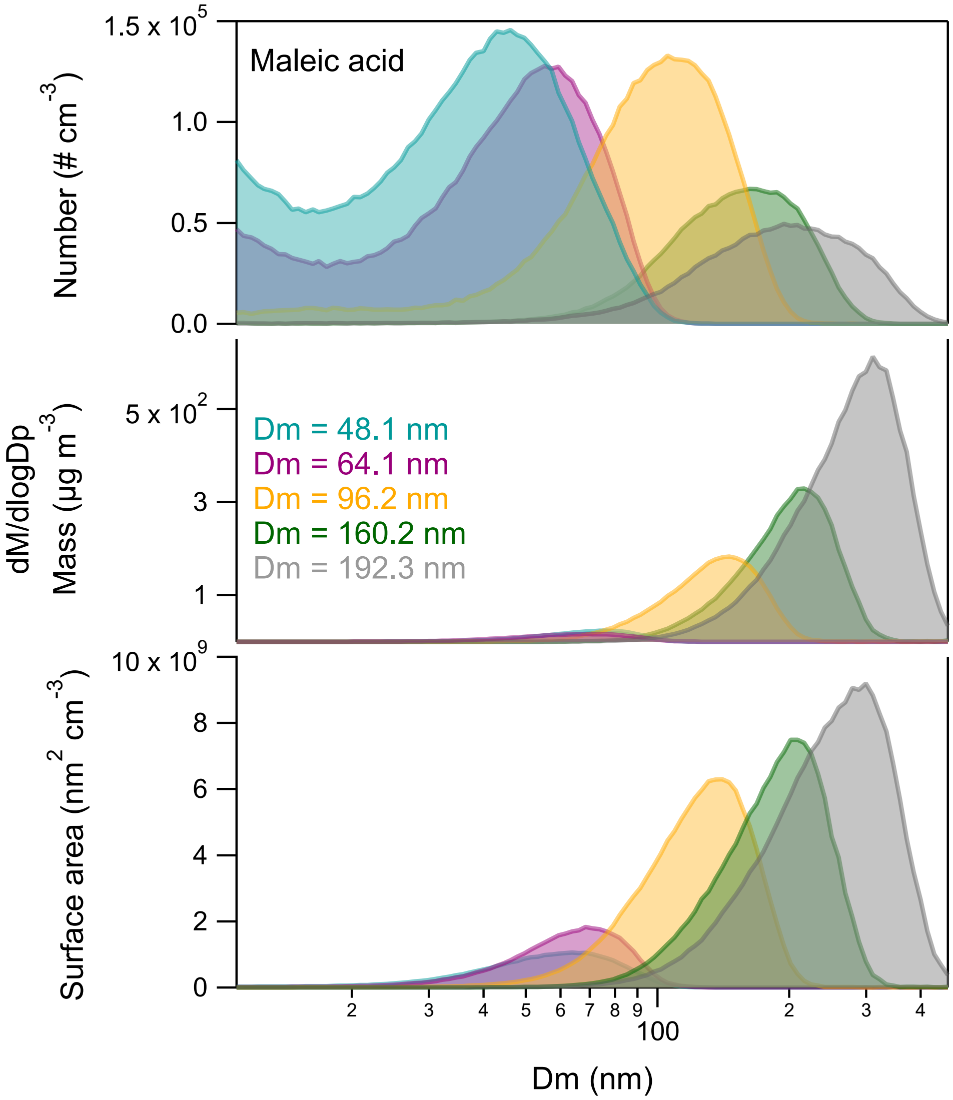

Fig. S13. Electrical mobility diameter distributions of maleic acid particles (size-resolved measurements). Concentration (top), mass (middle) and surface (bottom)particle size distribution of maleic acid. Different colors represent different sizes. The selected size is given as an inset.

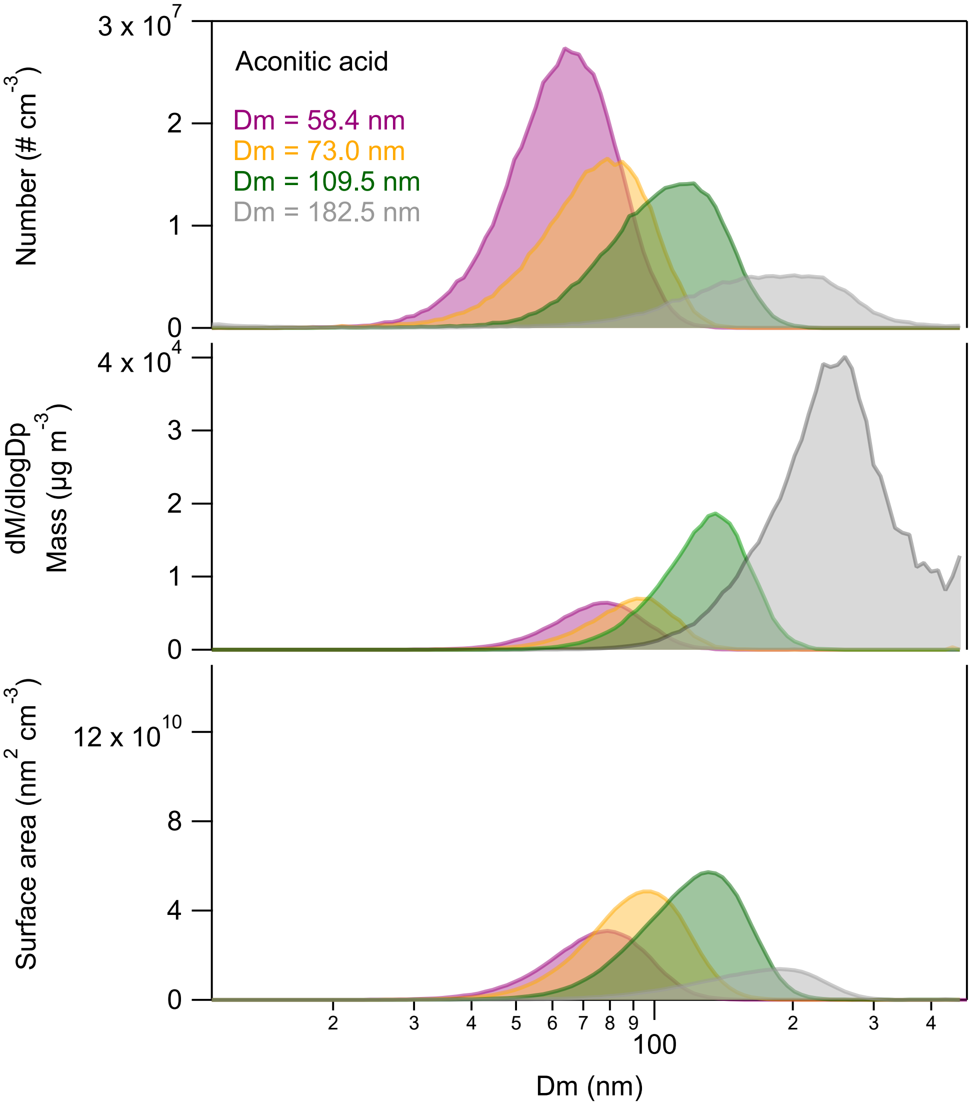

Fig. S14. Electrical mobility diameter distributions of aconitic acid particles (size-resolved measurements). Concentration (top), mass (middle) and surface (bottom) particle size distribution of aconitic acid. Different colors represent different sizes. The selected size is given as an inset.

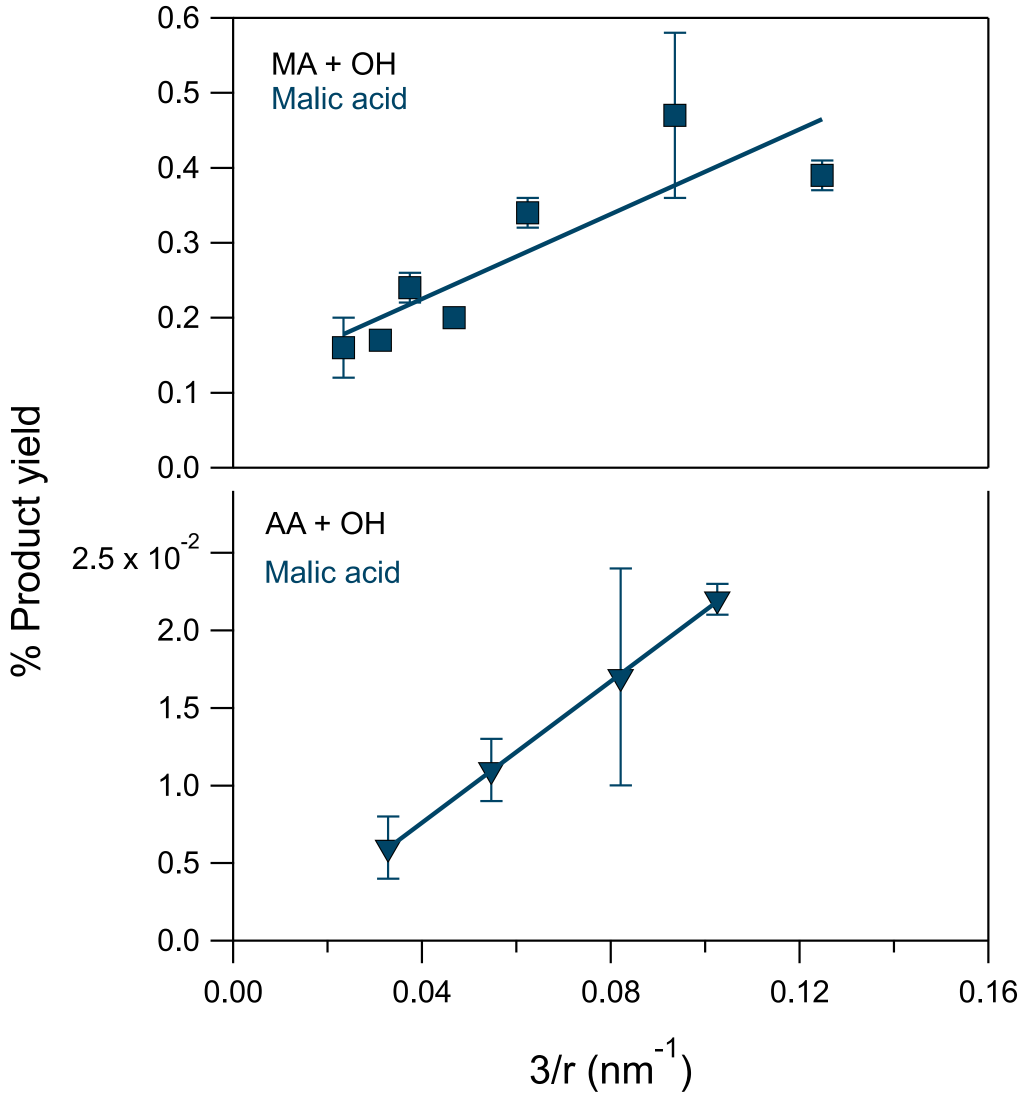

Fig. S15. Product yields at various particle sizes (offline measurements). Malic acid (MA+OH: blue squares, AA+OH: blue triangles) formation yields as a function of the particle surface-to-volume ratio. The radius is based on the electrical mobility diameter.

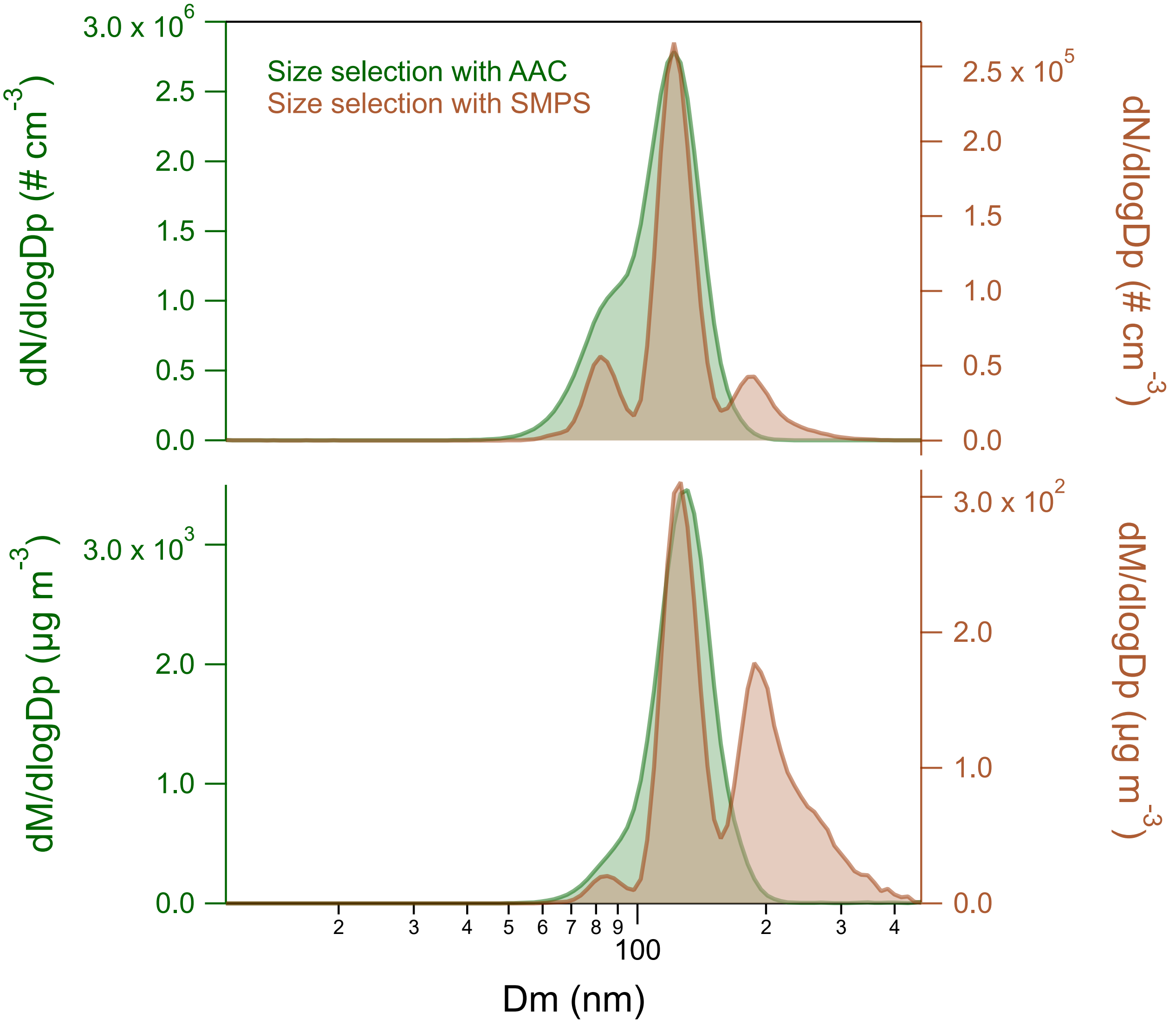

S

Fig. S16. Electrical mobility diameter distributions of monodisperse citric acid particles. CA particle size distribution of d = 108.1 nm, selected by AAC (green / left axis) and DMA (brown / right axis).

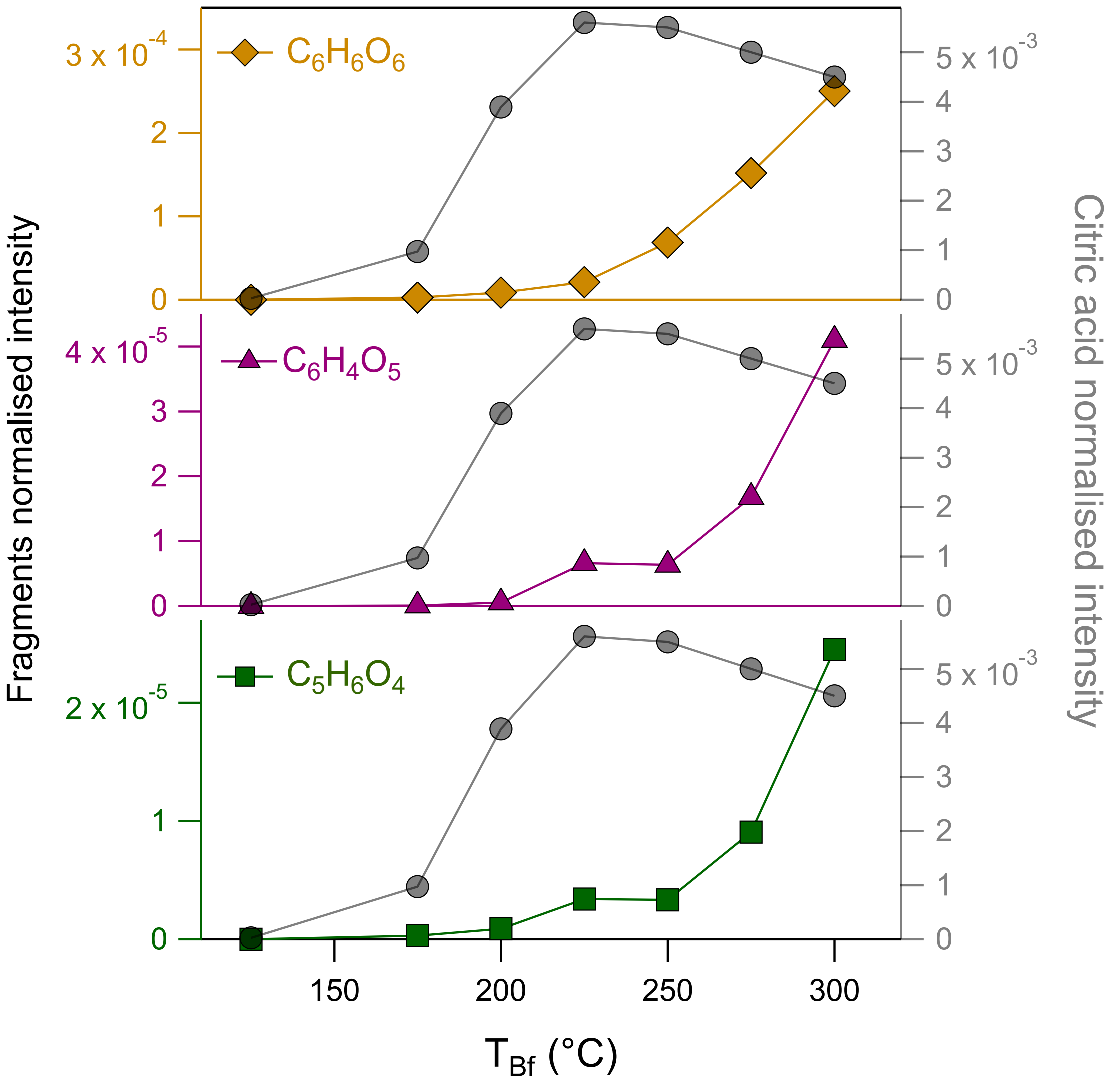

Fig. S17. Fragments of citric acid at various temperatures of WALL-E inlet. Normalized intensities of CA (gray circles), $C_6H_6O_6$ (yellow diamonds), $C_6H_4O_5$ (purple triangles) and $C_5H_6O_4$ (green squares) as a function of temperature.

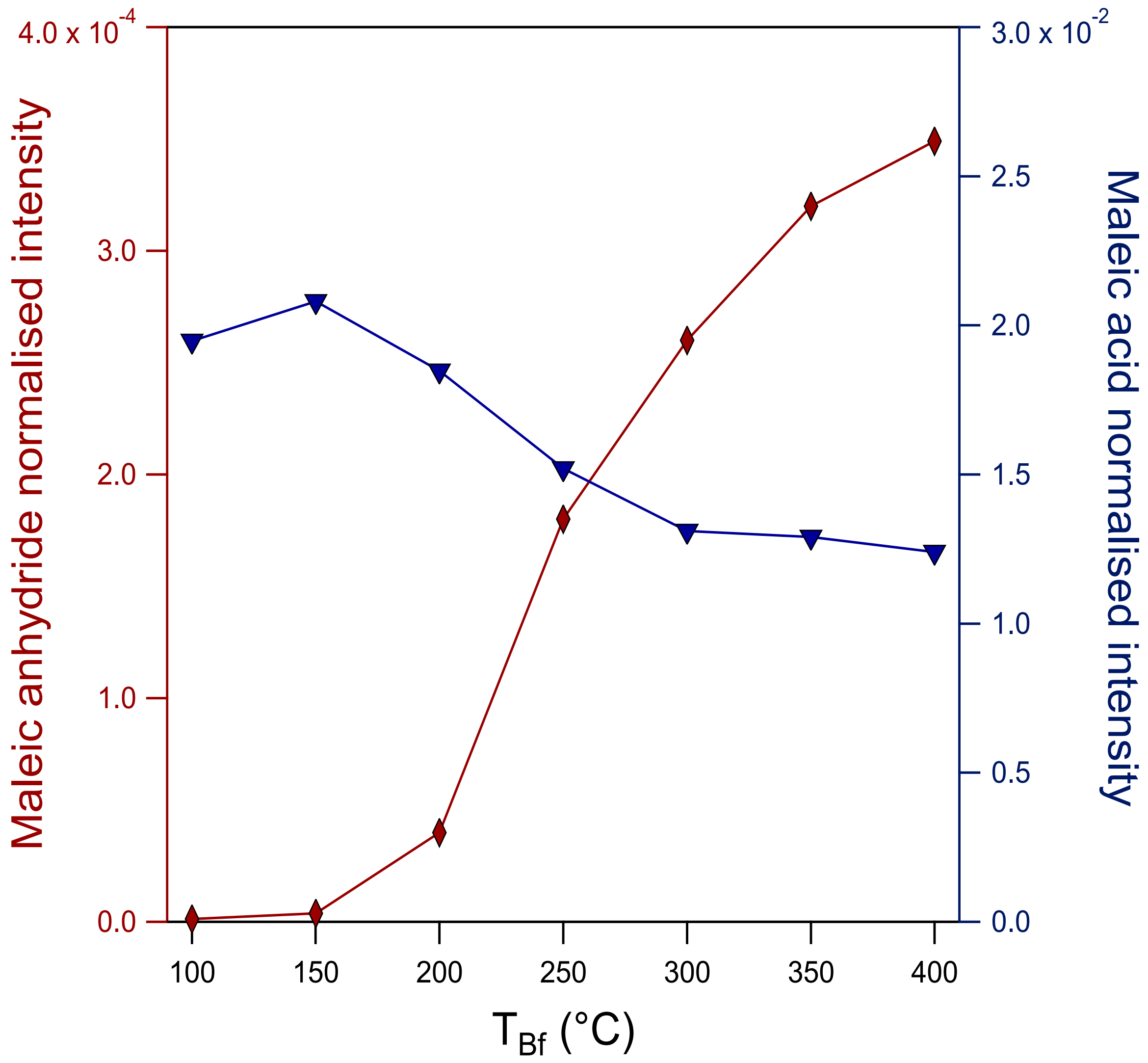

Fig. S18. Fragments of maleic acid at various temperatures of WALL-E inlet. Normalized intensities of MA (blue diamonds) and maleic anhydride ($C_4H_2O_3$, red diamonds) as a function of temperature.

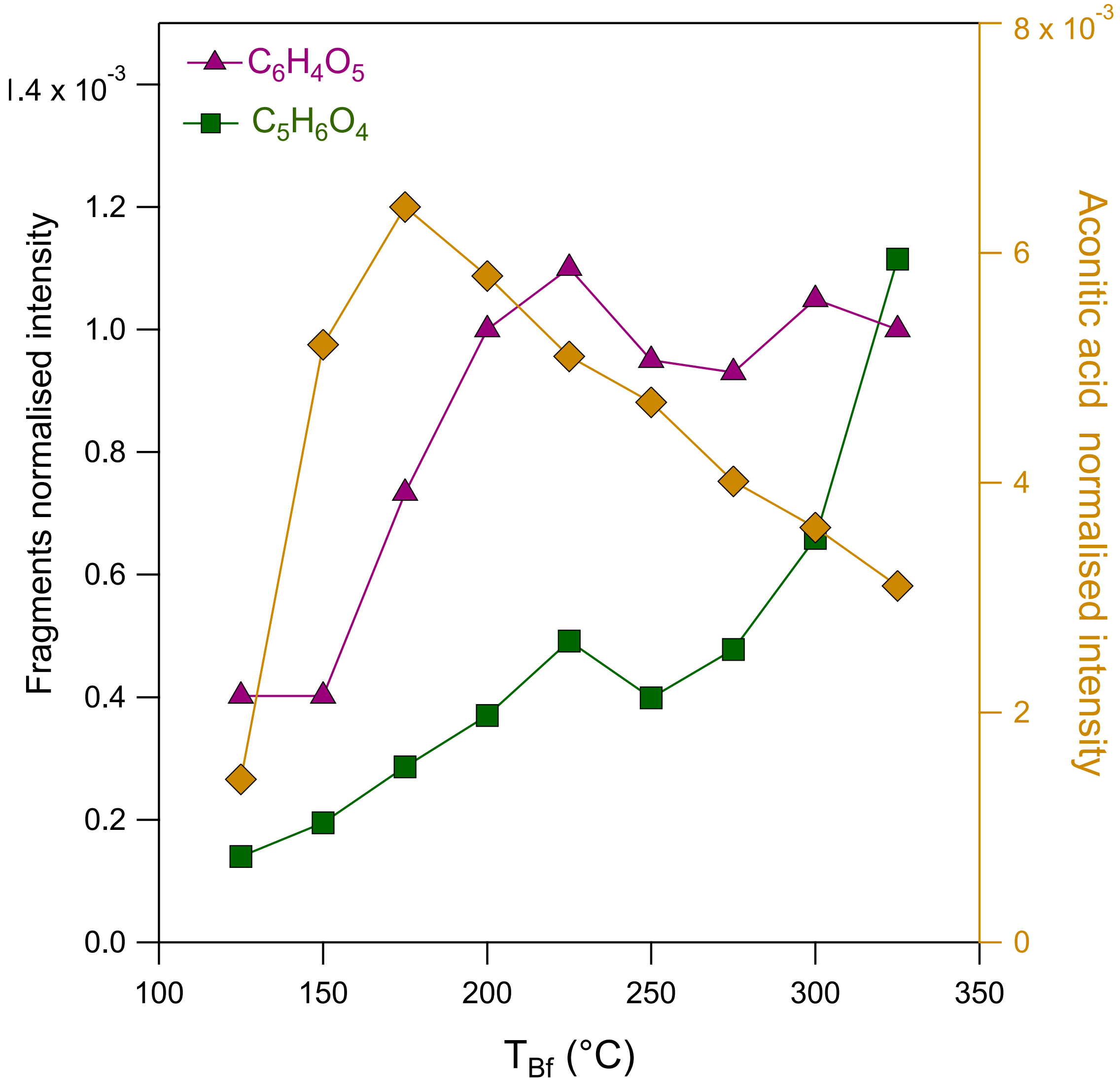

Fig. S19. Fragments of aconitic acid at various temperatures of WALL-E inlet. Normalized intensities AA (yellow diamonds), $C_6H_4O_5$ (purple triangles) and $C_5H_6O_4$ (green squares) as a function of temperature.

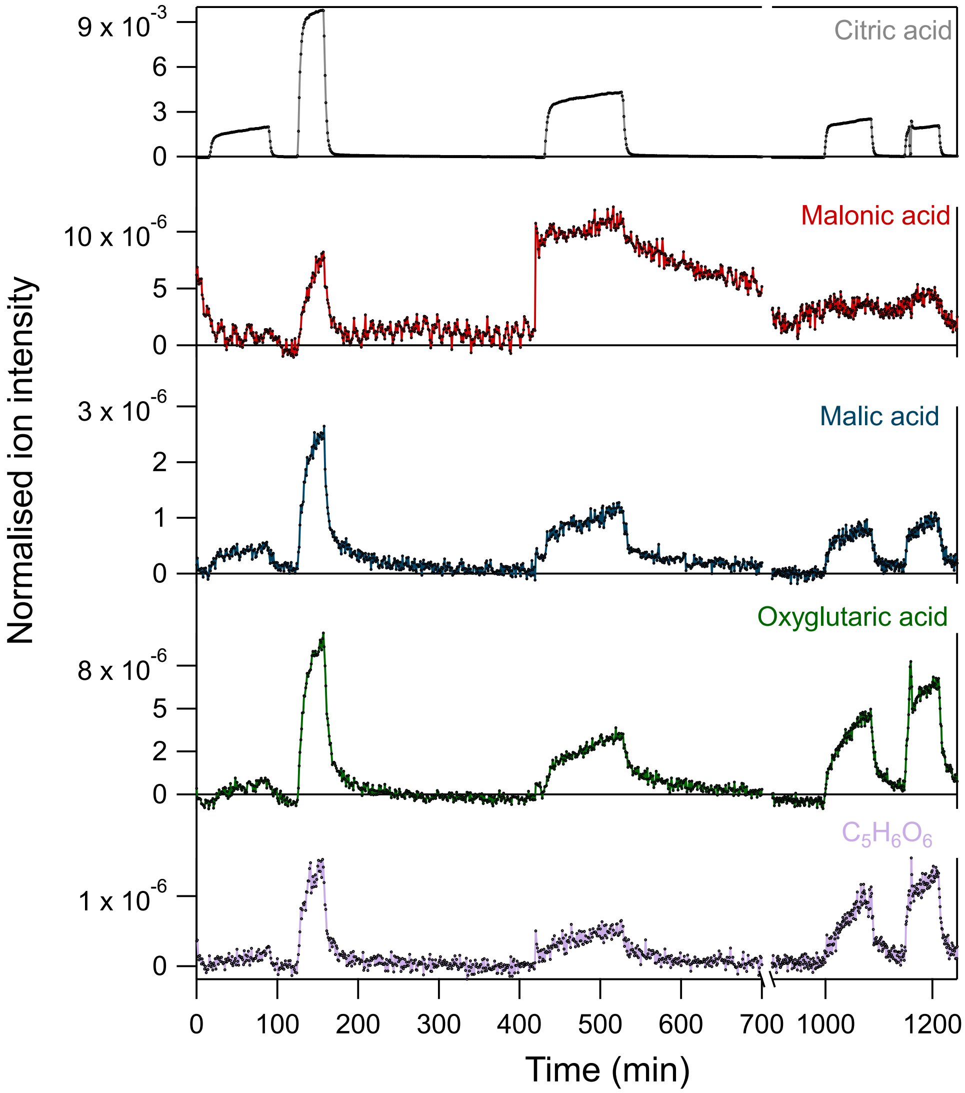

Fig. S20. Time series of citric acid and detected products. Citric, malonic, malic and oxyglutaric acid, and $C_5H_6O_6$ intensities recorded at different sizes as a function of time. Selected sizes: 300 nm (0 – 100 min), 105 nm (100 – 200 min), 147 nm (400 – 600 min), 85 nm (900 – 1100 min) and 85 nm (1100 – 1250 min).

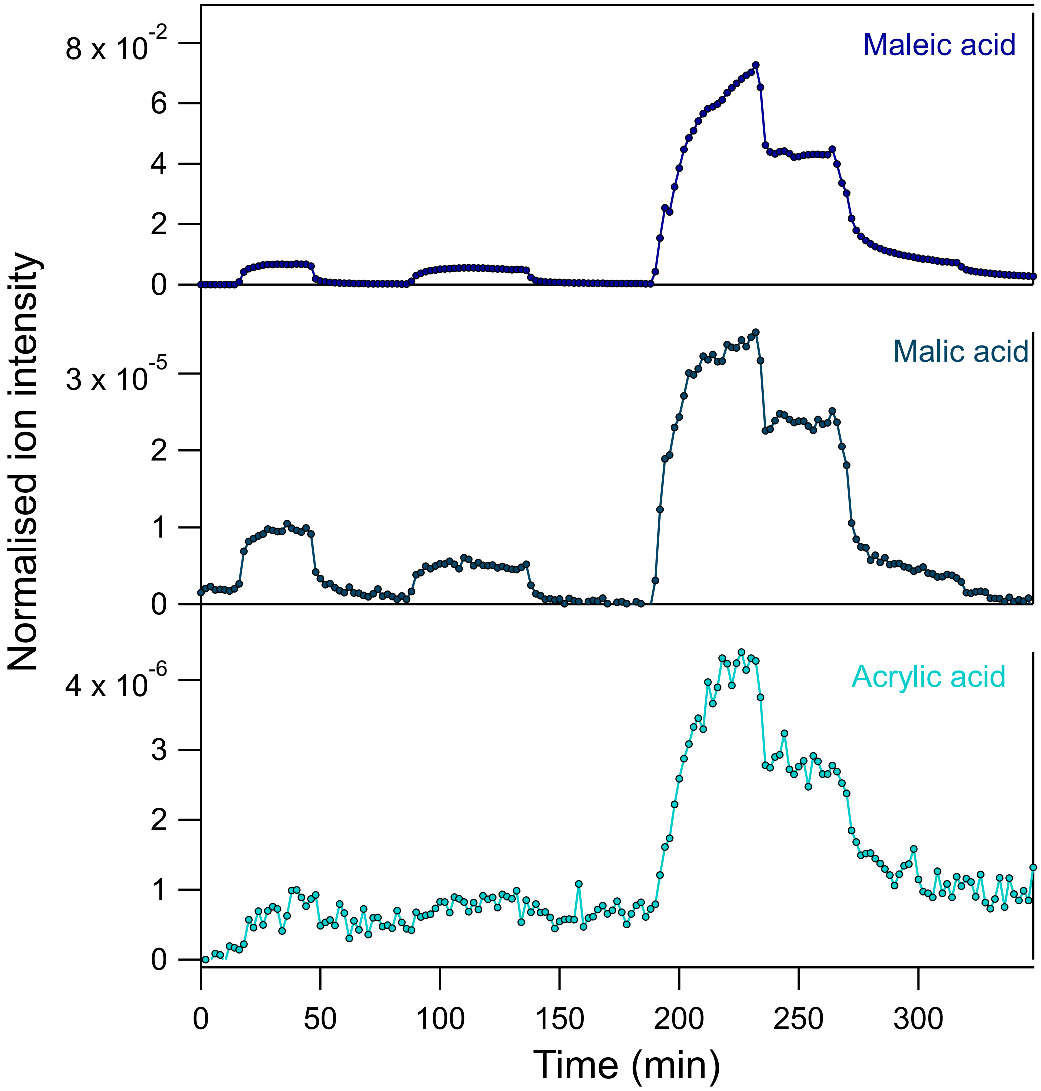

Fig. S21. Time series of maleic acid and detected products. Maleic, malic and acrylic acid intensities recorded at different sizes as a function of time. Selected sizes: 75 nm (0 – 75 min), 100 nm (80 – 150 min), 200 nm (170 – 230 min) and 150 nm (230 – 300 min).

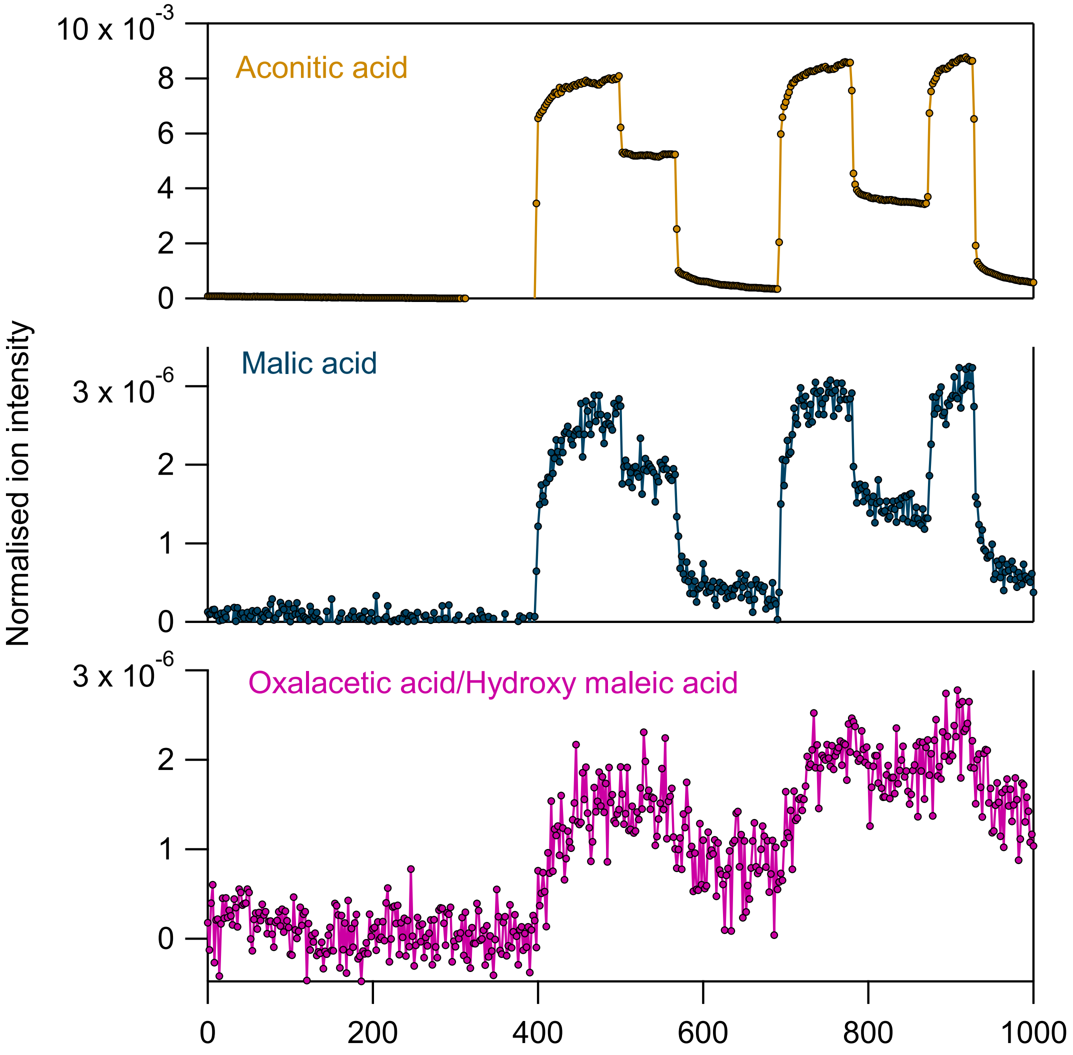

Fig. S22. Time series of aconitic acid and detected products. Aconitic, malic and oxalacetic/hydroxy maleic acid intensities recorded at different sizes as a function of time. Selected sizes: 250 nm (400 – 500 min), 150 nm (500 – 600 min), 250 nm (700 – 780 min), 100 nm (780 – 880 min) and 250 (880 – 930 min).

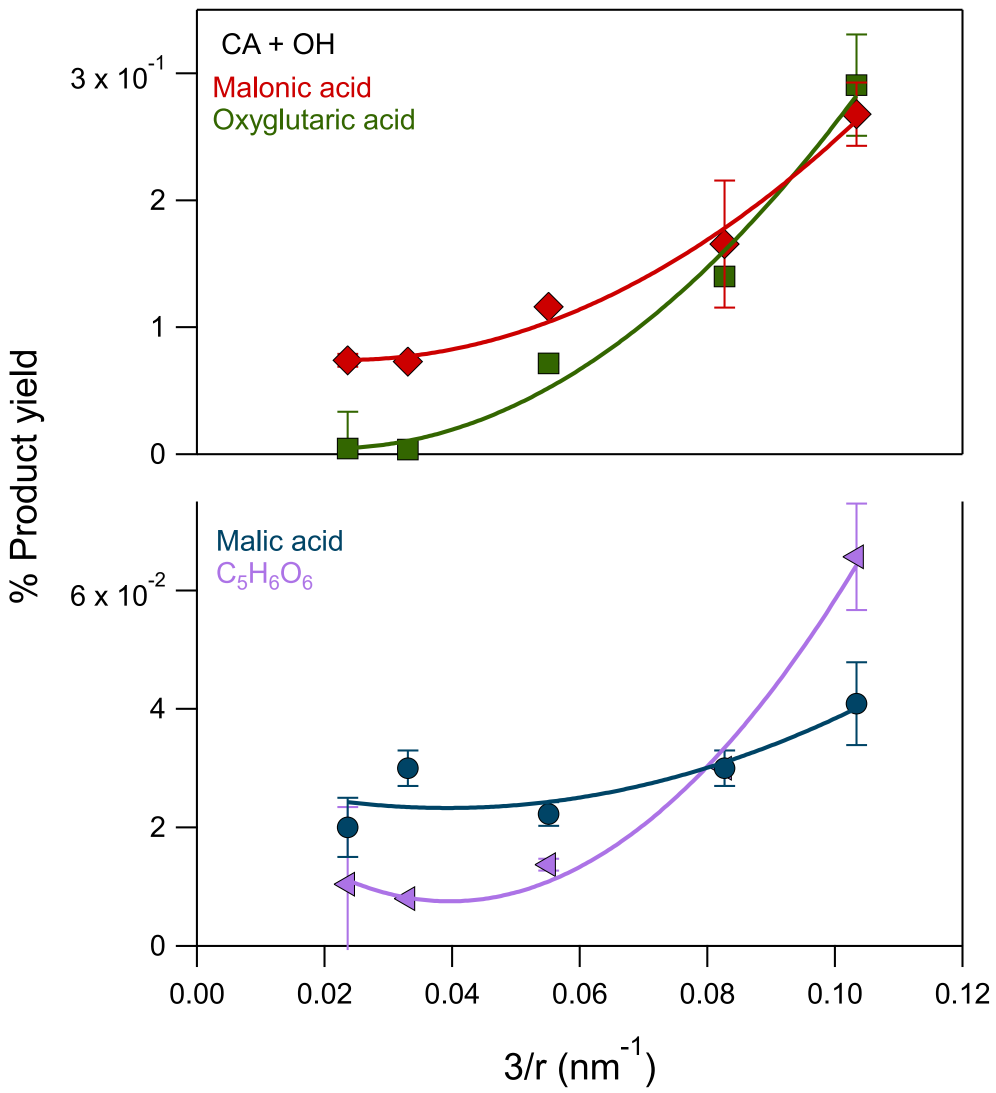

Fig. S23. **Product yields at various citric acid particle sizes (online measurements).** Malonic (red diamonds), oxyglutaric (green squares), malic (blue circles) acid and $C_5H_6O_5$ (purple triangles) formation yields from citric acid particle oxidation as a function of the particle surface-to-volume ratio (3/radius). The radius is based on the electrical mobility diameter.

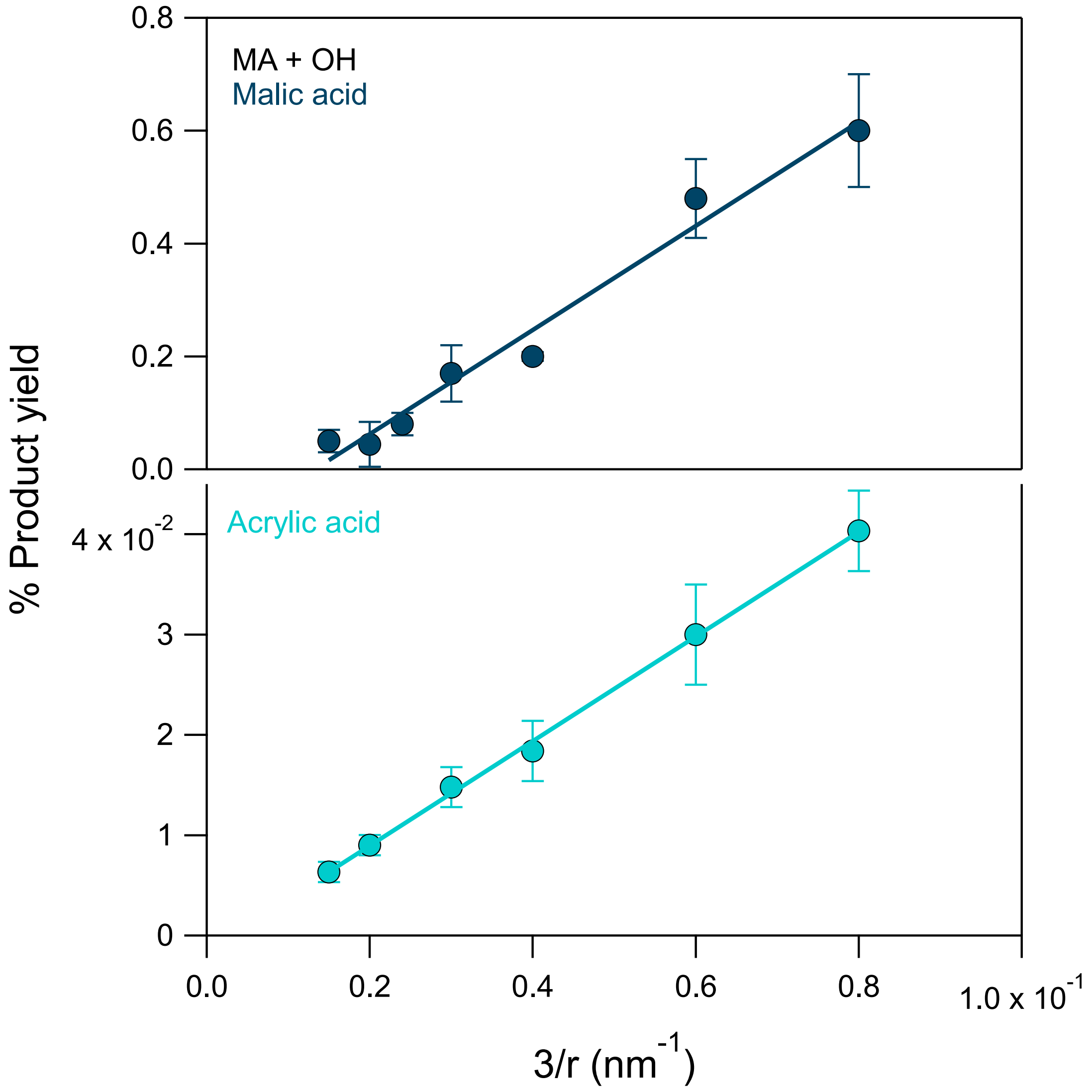

Fig. S24. Product yields at various maleic acid particle sizes (online measurements). Malic (blue circles) and acrylic (light blue circles) acid formation yields from maleic acid particle oxidation as a function of the particle surface-to-volume ratio (3/radius). The radius is based on the electrical mobility diameter.

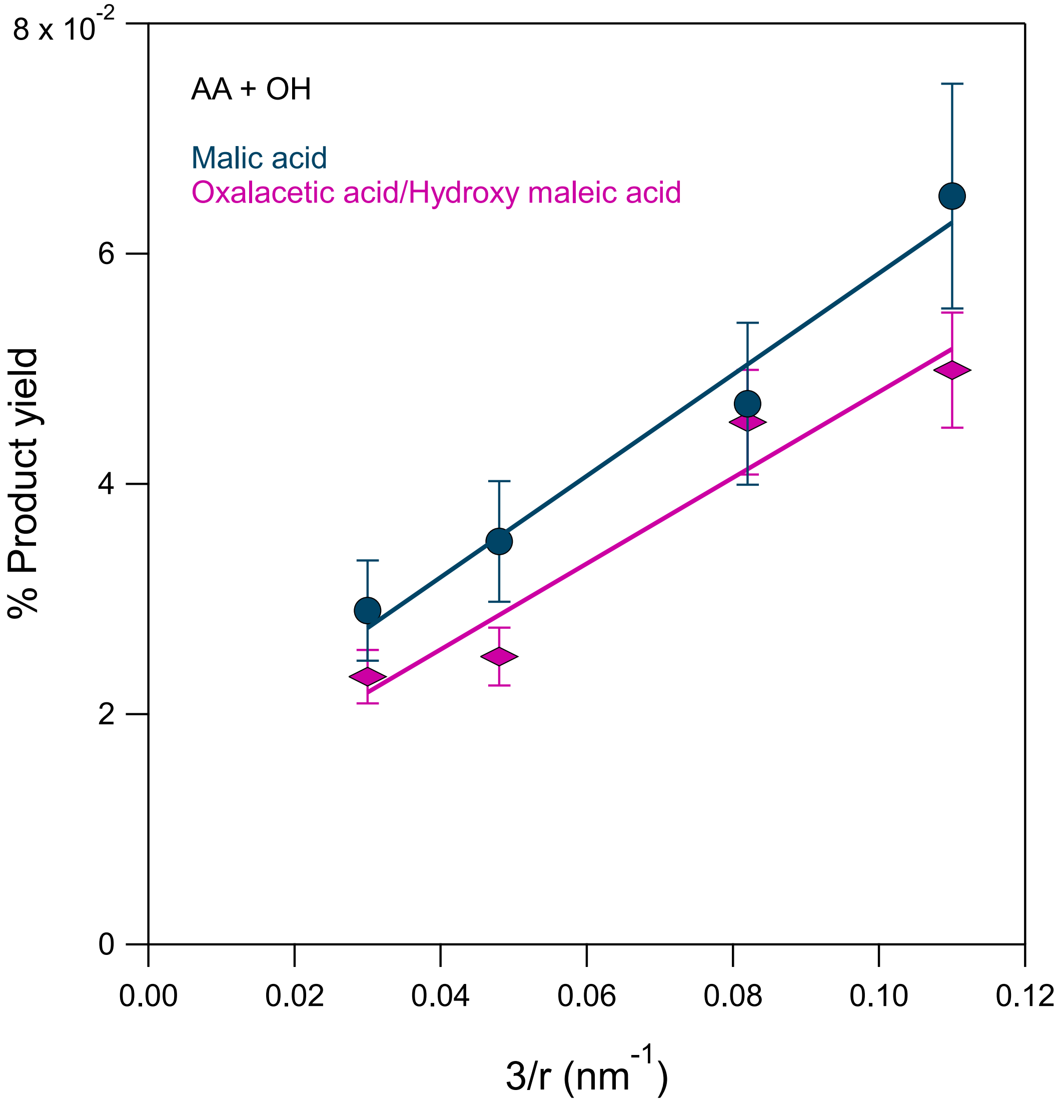

Fig. S25. Product yields at various aconitic acid particle sizes (online measurements). Malic (blue circles) and oxalacetic/hydroxy maleic (pink diamonds) acid formation yields from aconitic acid particle oxidation as a function of the particle surface-to-volume ratio (3/radius). The radius is based on the electrical mobility diameter.

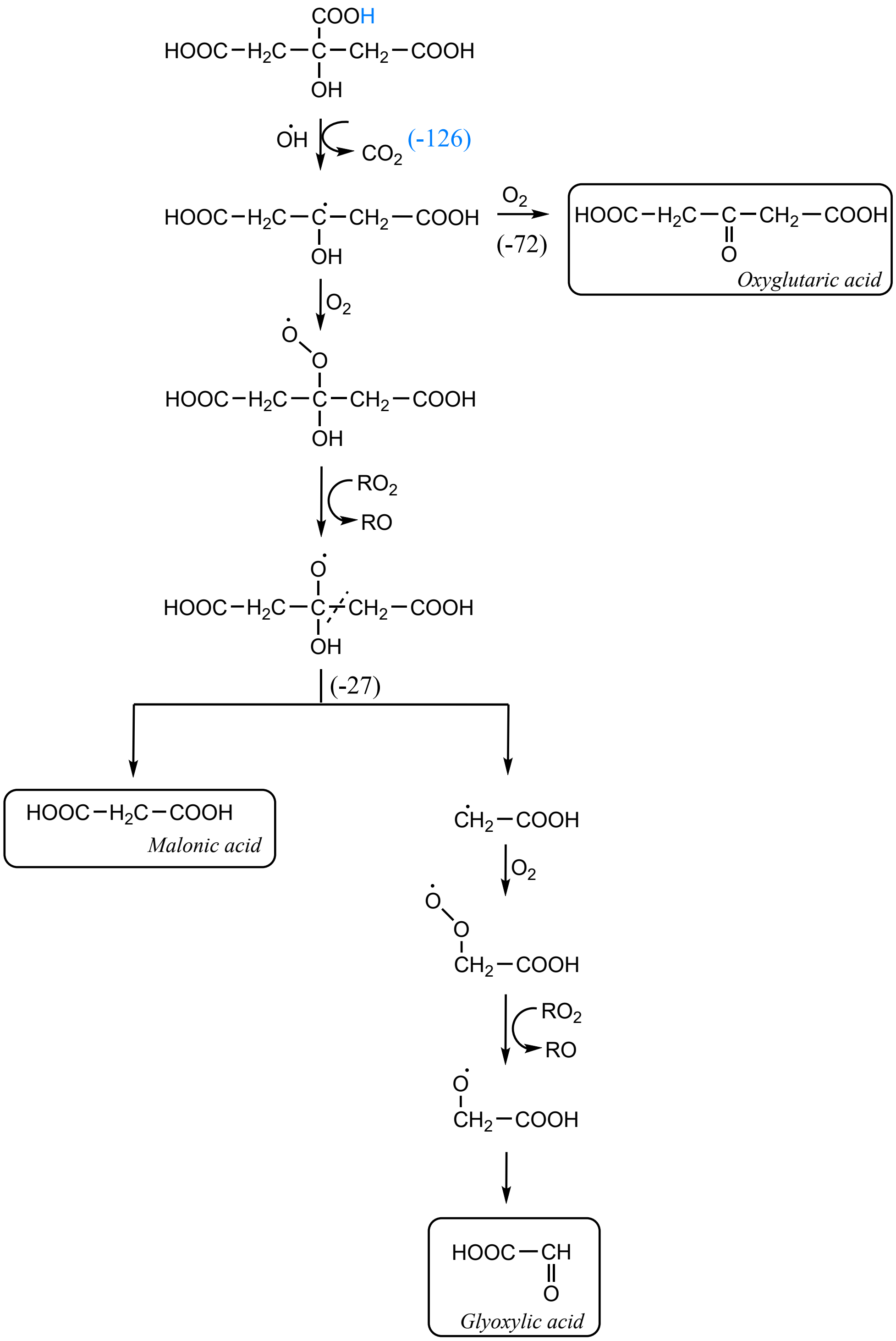

Fig. S26a. Proposed mechanistic scheme for the reaction of OH radical with citric acid: middle carboxylic H abstraction. Quantities in parentheses are the DFT energetics of each path (kJ mol^{-1}) computed at the ωB97X-V/TZVPPD level. The experimentally identified products are given inside a solid box.

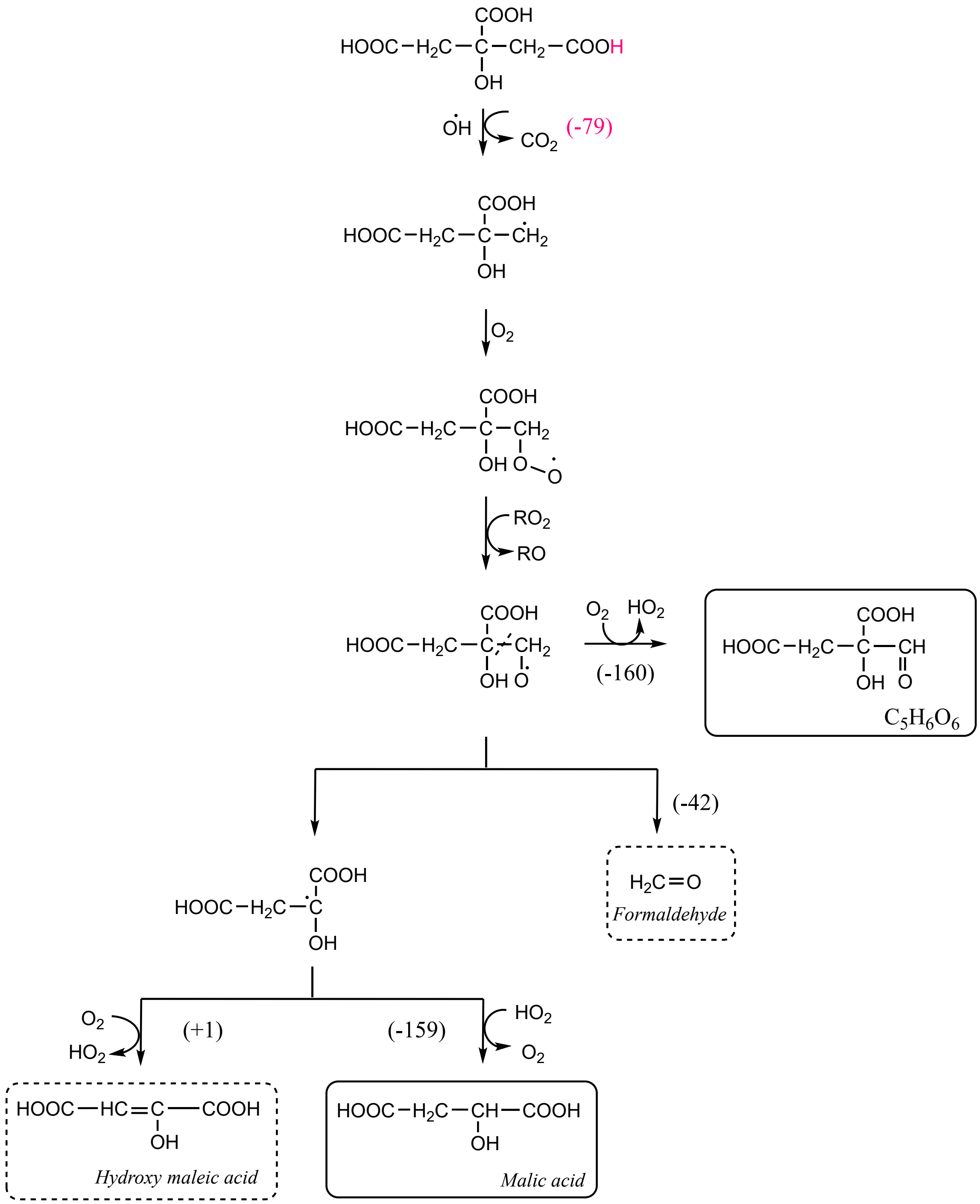

Fig. S26b. Proposed mechanistic scheme for the reaction of OH radical with citric acid: side carboxylic H abstraction. Quantities in parentheses are the DFT energetics of each path ($kJ\ mol^{-1}$) computed at the ωB97X-V/TZVPPD level. The experimentally identified products are given inside a solid box.

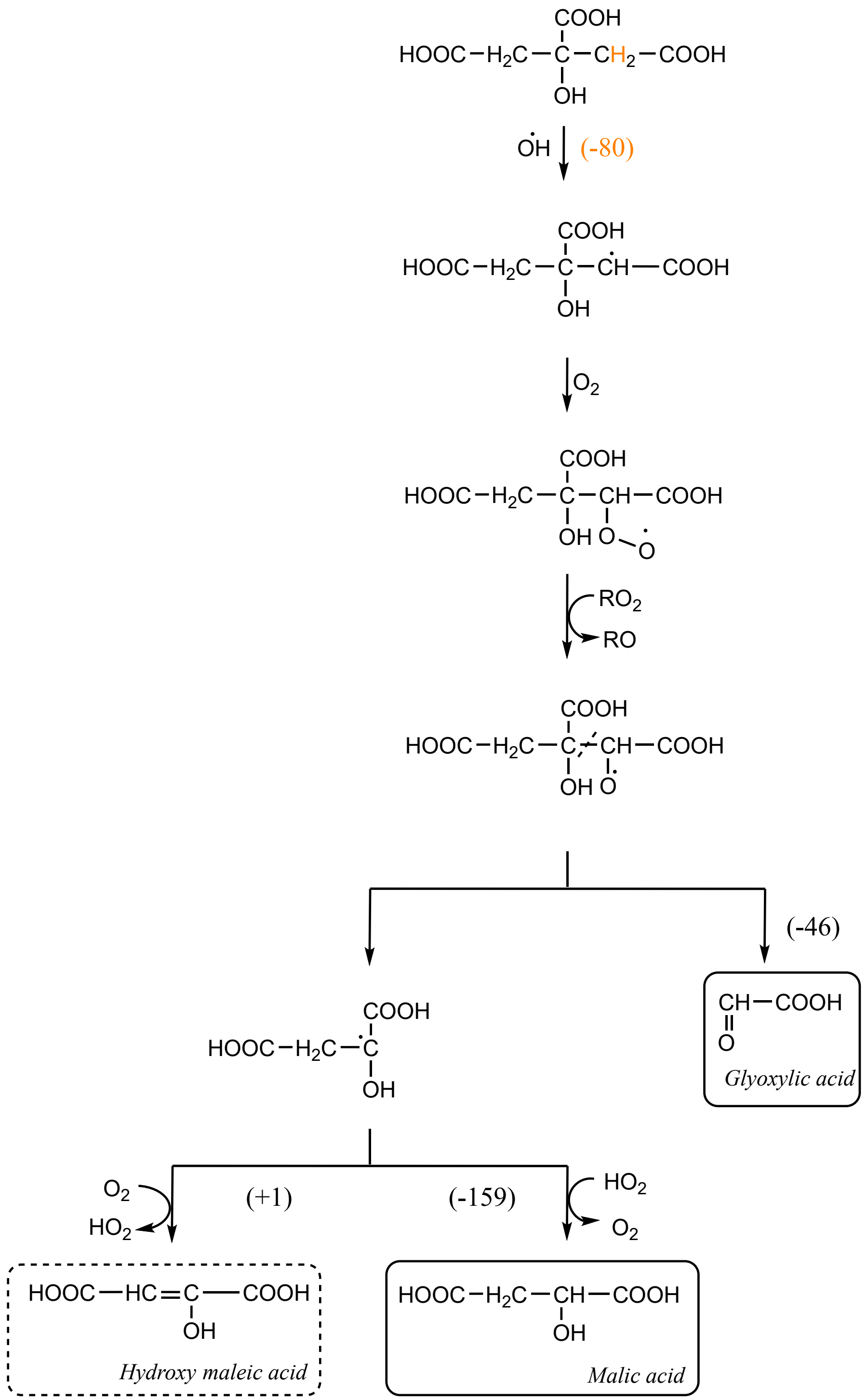

Fig. S26c. **Proposed mechanistic scheme for the reaction of OH radical with citric acid: –CH_2 group H abstraction.** Quantities in parentheses are the DFT energetics of each path (kJ mol^{-1}) computed at the ωB97X-V/TZVPPD level. The experimentally identified products are given inside a solid box.

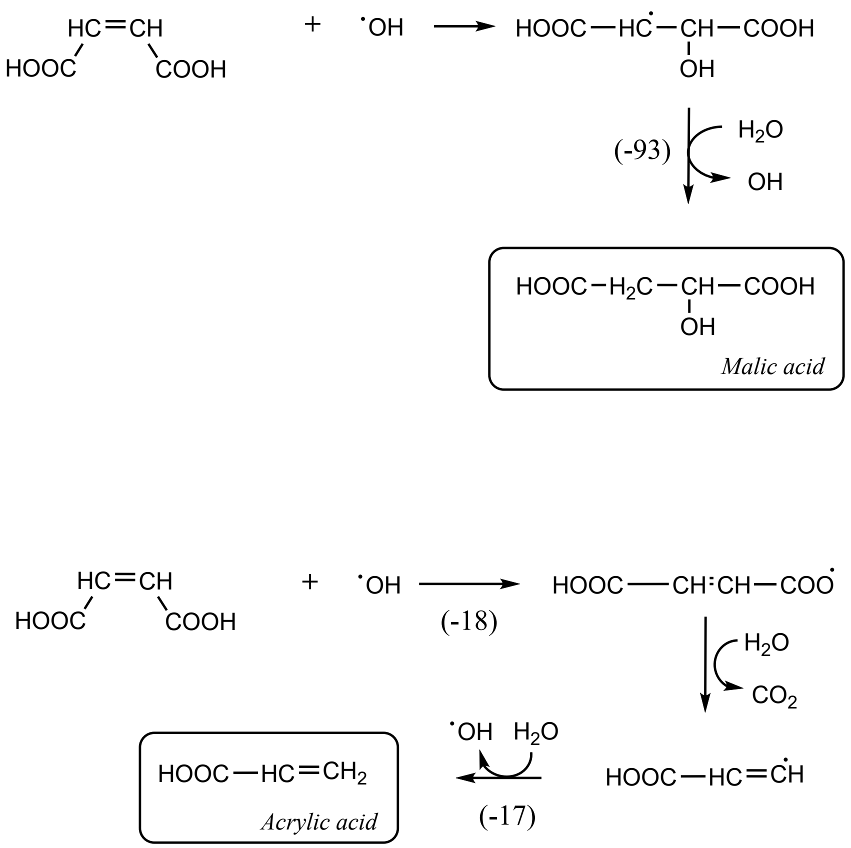

Fig. S27. Proposed mechanistic scheme for the reaction of OH radical with maleic acid. a. OH addition b. H abstraction. Quantities in parentheses are the DFT energetics of each path (kJ mol^{-1}) computed at the ωB97X-V/TZVPPD level. The experimentally identified products are given inside a solid box.

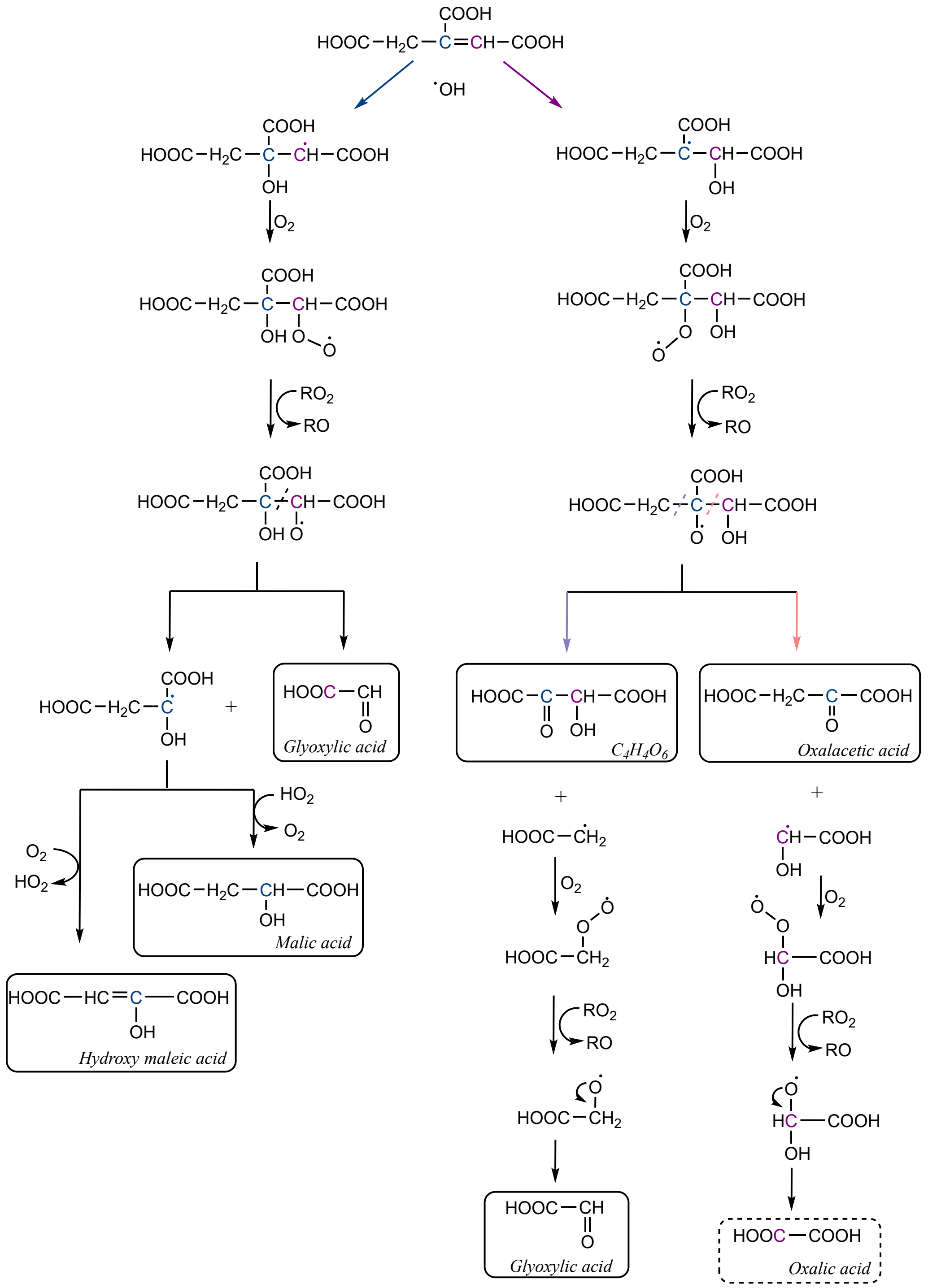

Fig. S28. Proposed mechanistic scheme for the reaction of OH radical with aconitic acid. The experimentally identified products are inside solid box.

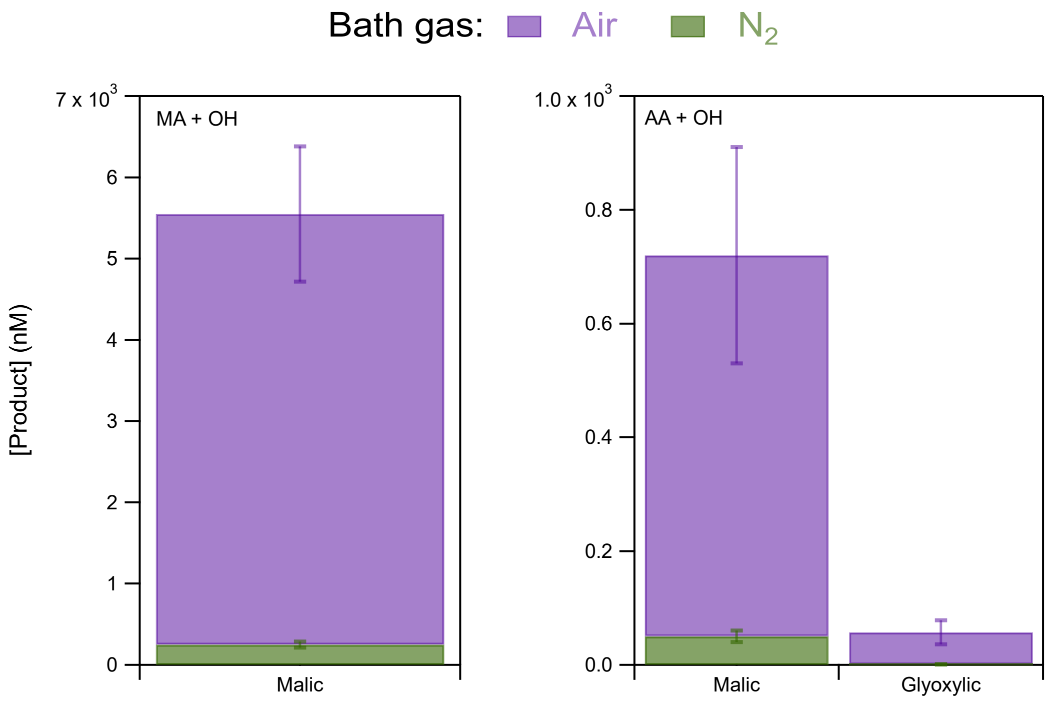

Fig. 29. Evaluation of the role of O_2: Comparison plot of the product concentrations in the presence (orange) and the absence (green) of O_2 for the reactions of maleic acid + OH and aconitic acid + OH.

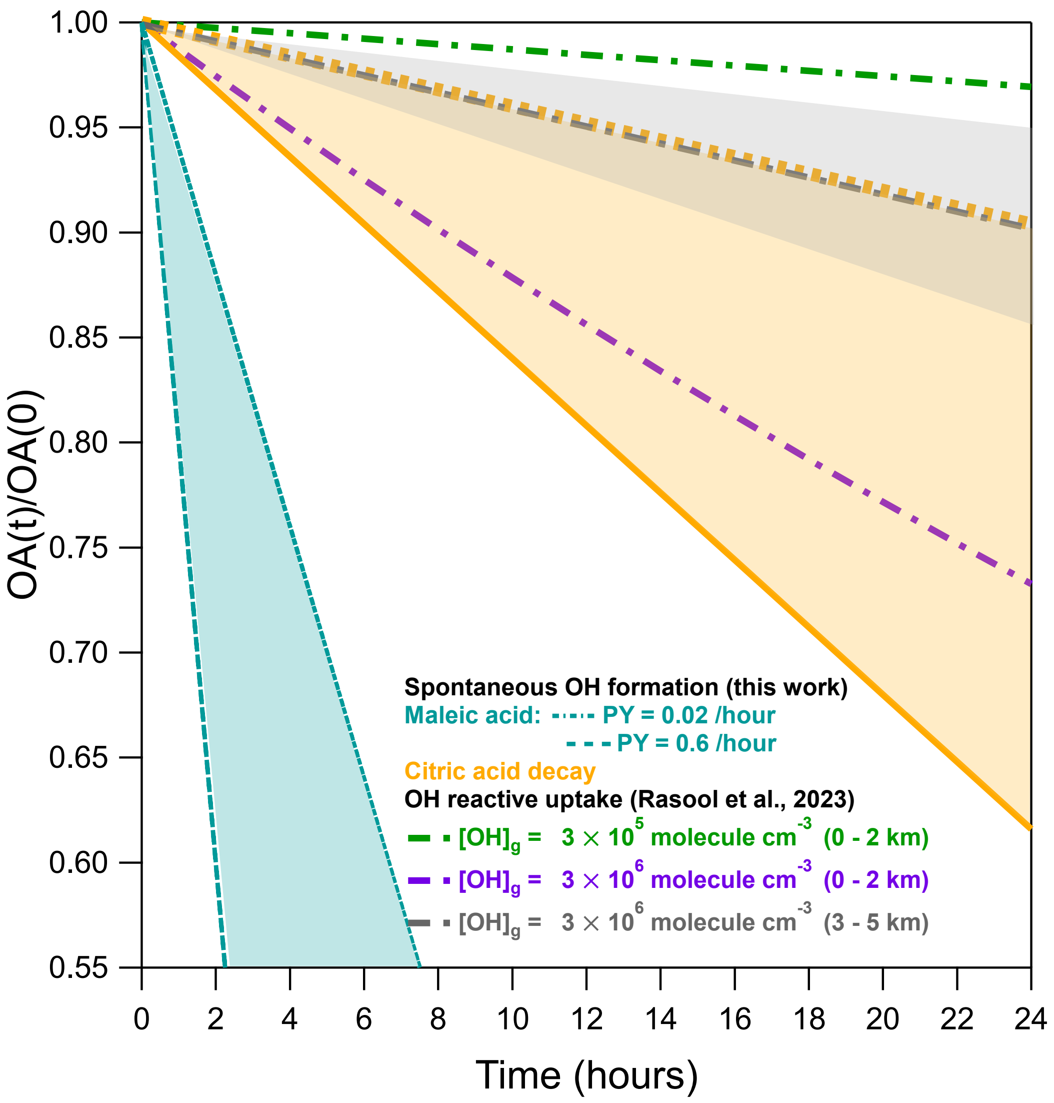

Fig. S30. Atmospheric impact. Citric (orange region) and maleic (light blue region) acid decay rates due to the spontaneous OH oxidation for the range of product yield of $(4–16)\times10^{-3}$ h^{-1} and $(0.6–2)\times10^{-1}$ h^{-1}. Organic aerosol decay due to the heterogeneous oxidation by OH uptake at a. $[OH]_g= 3\times(10^5–10^6)$ molecules cm^{-3}, at altitudes 0–2 km (green and purple) and b: at $[OH]_g= 3\times10^6$ molecules cm^{-3}, at altitudes 3–5 km and k_{uptake} of $(2–6)\times10^{-13}$ cm^3 $molecules^{-1}$ s^{-1} (gray region (gray solid line is the mean value).

Table S1: Compounds from the reaction of OH radicals with CA, detected using LC-HRMS (offline measurements) and WALL-E Vocus AIM (online measurements).

		LC/MS		Vocus AIM	
		**Formula**	**Mass**	**Formula**	**Mass**
HCOO-CH2-C(OH)(COOH)-CH2-COOH	Citric acid	$[C_6H_7O_7]^-$	191.01973	$[C_6H_8IO_7]^-$	
O = CH-COOH	Glyoxylic acid	$[C_2HO_3]^-$	72.99312	$[C_2H_2IO_3]^-$	200.90541
HCOO-CH2-COOH	Malonic acid	$[C_3H_3O_4]^-$	103.00368	$[C_3H_4IO_4]^-$	230.91598
HCOO-CH2-CH(OH)-COOH	Malic acid	$[C_4H_5O_5]^-$	133.01425	$[C_4H_6IO_5]^-$	260.92654
HCOO-CH=C(OH)-COOH	Hydroxy maleic acid	–	–	$[C_4H_4IO_5]^-$	258.91089
HCOO-CH2-C(O)-CH2-COOH	Oxyglutaric acid	–	–	$[C_5H_6IO_5]^-$	272.92654
HCOO-CH2-C(OH)(COOH)-CH=O	$C_5H_6O_6$	–	–	$[C_5H_6IO_6]^-$	288.92146

Table S2: Compounds from the reaction of OH radicals with MA, detected using LC-HRMS (offline measurements) and WALL-E Vocus AIM (online measurements).

		LC/MS		Vocus AIM	
		**Formula**	**Mass**	**Formula**	**Mass**
HCOO-CH=CH-COOH	Maleic acid	$[C_4H_3O_4]^-$	115.00368	$[C_4H_4IO_4]^-$	242.91598
HCOO-CH2-CH(OH)-COOH	Malic acid	$[C_4H_5O_5]^-$	133.01425	$[C_4H_6IO_5]^-$	260.92654
CH2=CH-COOH	Acrylic acid	–	–	$[C_3H_4IO_2]^-$	198.92615

Table S3: Compounds from the reaction of OH radicals with AA, detected using LC-HRMS (offline measurements) and WALL-E Vocus AIM (online measurements).

		LC/MS		Vocus AIM	
		**Formula**	**Mass**	**Formula**	**Mass**
HCOO-CH2-C(COOH)=CH-COOH	Aconitic acid	$[C_6H_5O_6]^-$		$[C_6H_6IO_6]^-$	
O = CH-COOH	Glyoxylic acid	$[C_2HO_3]^-$	72.99312	$[C_2H_2IO_3]^-$	200.90541
HCOO-CH2-COOH	Malonic acid	$[C_3H_3O_4]^-$	103.00368	$[C_3H_4IO_4]^-$	230.91598
HCOO-CH2-CH(OH)-COOH	Malic acid	$[C_4H_5O_5]^-$	133.01425	$[C_4H_6IO_5]^-$	260.92654
HCOO-CH=C(OH)-COOH	Hydroxy maleic acid	–	–	$[C_4H_4IO_5]^-$	258.91089
HCOO-CH2-C(O)-COOH	Oxalacetic acid	–	–	$[C_4H_4IO_5]^-$	258.91089
HCOO-C(O)-CH(OH)-(COOH)	$C_4H_4O_6$	–	–	$[C_4H_4IO_6]^-$	274;905808

Table S4: Experimental conditions and LC-HRMS data of humidity dependent experiments performed with citric acid.

[Citric acid] [a,b]	Mass [c]	Median size [d]	[Malonic acid] [a,b]	[Malic acid] [a,b]	[Glyoxilic acid] [a,b]
**RH = 0 %**					
4715 ± 222		94.7	-	0.032 ± 0.002	-
				**0.032 ± 0.002**	
**RH = 10 %**					
4610 ± 143	3200	98.2	0.29 ± 0.02	0.050 ± 0.002	-
4200 ± 195	3278		0.30 ± 0.01	0.10 ± 0.01	-
			**0.30 ± 0.05** [e]	**0.08 ± 0.03** [e]	**-**
**RH = 32 %**					
2216 ± 289	4030	98.2	0.58 ± 0.02	0.10 ± 0.01	0
2495 ± 88	3854		0.54 ± 0.01	0.15 ± 0.01	0.11 ± 0.01
2622 ± 73	3778		0.64 ± 0.04	0.22 ± 0.01	0.10 ± 0.01
2688 ± 146	4120		0.62 ± 0.06	0.18 ± 0.03	0.15 ± 0.01
			**0.60 ± 0.05** [e]	**0.16 ± 0.06** [e]	**0.12 ± 0.03** [e]
**RH = 55 %**					
2256 ± 178	3254		0.45 ± 0.01	0.29 ± 0.01	0.16 ± 0.01
2136 ± 201	3200	105.5	0.65 ± 0.01	0.10 ± 0.01	0.15 ± 0.01
2006 ± 99	3210		0.69 ± 0.03	0.20 ± 0.01	0.17 ± 0.03
			**0.60 ± 0.15** [e]	**0.20 ± 0.1** [e]	**0.16 ± 0.01** [e]
**RH = 75 %**					
2955 ± 138	3564		0.81 ± 0.07	0.27 ± 0.03	0.14 ± 0.01
2885 ± 133	3500	113.4	0.86 ± 0.09	0.29 ± 0.06	0.21 ± 0.04
2921 ± 84	3478		1.04 ± 0.09	0.27 ± 0.02	0.20 ± 0.01
			**0.90 ± 0.14** [e]	**0.28 ± 0.01** [e]	**0.18 ± 0.04** [e]
**RH = 99 %**					
1824 ± 142	4125	113.4	0.95 ± 0.04	0.37 ± 0.02	-
2390 ± 113	4154		0.97 ± 0.06	0.39 ± 0.01	0.22 ± 0.01
			**0.96 ± 0.01** [e]	**0.38 ± 0.02** [e]	**0.22 ± 0.02** [e]

[a]Reactant and products concentration in µM. The sample of each experiment was analyzed 3 times and the reported value represents the mean value; [b]Errors represent the standard deviation; [c]Particle mass in µg m^{-3}; [d]Median particle diameter in nm; [e]Mean value of the products concentration for each experimental condition.

Table S5: Experimental conditions and LC-HRMS data of humidity dependent experiments performed with maleic acid.

[Maleic acid] [a,b]	Mass [c]	Median size [d]	[Malic acid] [a,b]
**RH = 0 %**			
4880 ± 128	2480	76.4	0.076 ± 0.002
			**0.076 ± 0.002** [e]
**RH = 10 %**			
4457 ± 59	3180	86.1	1.08 ± 0.09
			**1.08 ± 0.09** [e]
**RH = 32 %**			
4280 ± 264	7280	105.5	3.39 ± 0.14
1299 ± 88	3400		2.50 ± 0.11
			**2.95 ± 0.45** [e]
**RH = 55 %**			
3652 ± 74	7830		5.30 ± 0.24
3652 ± 201	8310	113.4	5.08 ± 0.18
4078 ± 215	8680		6.41 ± 0.29
			**5.60 ± 0.81** [e]
**RH = 75 %**			
4163 ± 96	9380		7.01 ± 0.34
4117 ± 243	11400	113.4	7.61 ± 0.11
2803 ± 155	7160		6.65 ± 0.47
			**7.09 ± 0.52** [e]
**RH = 99 %**			
1716 ±	18300	126.3	8.27 ± 0.46
2793 ±	23800		7.49 ± 0.74
2644 ±	10600		6.62 ± 0.77
			**7.46 ± 0.81** [e]

[a]Reactant and products concentration in μM. The sample of each experiment was analyzed 3 times and the reported value represents the mean value; [b]Errors represent the standard deviation; [c]Particle mass in μg m^{-3}; [d]Median particle diameter in nm; [e]Mean value of the products concentration for each experimental condition.

Table S6: Experimental conditions and LC-HRMS data of humidity dependent experiments performed with trans-aconitic acid.

[Aconitic acid] [a,b]	Mass [c]	Median size [d]	[Malic acid] [a,b]	[Glyoxylic acid] [a,b]
**RH = 0 %**				
5050 ± 140	25000	105.5	0.033 ± 0.002	-
			**0.033 ± 0.02** [e]	-
**RH = 10 %**				
4905 ± 28	33900	108.5	0.068 ± 0.004	0.030 ± 0.001
5942 ± 132	33180		0.089 ± 0.005	0.014 ± 0.002
			**0.078 ± 0.011** [e]	**0.022 ± 0.008** [e]
**RH = 32 %**				
2650 ± 50	43200	113.4	0.43 ± 0.04	0.064 ± 0.003
4753 ± 111	41258		0.41 ± 0.02	0.072 ± 0.007
			**0.42 ± 0.01** [e]	**0.068 ± 0.010** [e]
**RH = 55 %**				
5250 ± 326	48200		0.45 ± 0.05	0.041 ± 0.02
5347 ± 288	47500	131.0	0.98 ± 0.08	0.069 ± 0.04
4900 ± 297			0.051 ± 0.004	
			**0.72 ± 0.27** [e]	**0.055 ± 0.014** [e]
**RH = 75 %**				
5312 ± 512	43200	145.9	1.14 ± 0.07	0.13 ± 0.02
			**1.15 ± 0.07** [e]	**0.13 ± 0.02** [e]
**RH = 99 %**				
4159 ± 28	58700	145.9	0.79 ± 0.01	0.22 ± 0.05
5050 ± 144	55500		1.12 ± 0.08	0.26 ± 0.01
5570 ± 255	56700		1.31 ± 0.04	0.29 ± 0.04
			**1.07 ± 0.24** [e]	**0.26 ± 0.03** [e]

[a]Reactant and products concentration in µM. The sample of each experiment was analyzed 3 times and the reported value represents the mean value; [b]Errors represent the standard deviation; [c]Particle mass in µg m^{-3}; [d]Median particle diameter in nm; [e]Mean value of the products concentration for each experimental condition.

Table S7: Experimental conditions and LCMS data of the size resolved measurements performed in citric acid particles at RH = 55%. Formation yields of the products and size data are displayed.

[Citric acid] [a,b]	[Malonic acid] [a,b]	[Malic acid] [a,b]	Mass [c]	Median diameter [d]	% $PY_{Malonic}$ [e,f]	% PY_{Malic} [e,f]
**Selected size: De = 60 nm/Dm = 44.1 nm**						
125 ± 4	0.050 ± 0.006	0.006 ± 0.0001	67		0.040 ± 0.001	0.005 ± 0.001
269 ± 8	-	0.020 ± 0.0010	68	49.6	-	0.007 ± 0.001
231 ± 7	0.097 ± 0.001	0.012 ± 0.002	64		0.042 ± 0.04	0.005 ± 0.001
					**0.041 ± 0.001** [g]	**0.006 ± 0.001** [g]
**Selected size: De = 75 nm/Dm = 55.1 nm**						
404 ± 5	0.126 ± 0.02	0.021 ± 0.001	206	61.5	0.031 ± 0.02	0.005 ± 0.001
338 ±10		0.014 ± 0.002	17			0.004 ± 0.001
					**0.031 ± 0.0029** [g]	**0.005 ± 0.001** [g]
**Selected size: De = 85 nm/Dm = 62.5 nm**						
657 ± 7	0.163 ± 0.05	0.029 ± 0.008	412	66.5	0.025 ± 0.02	0.004 ± 0.001
805 ± 12	0.243 ± 0.04	0.034 ± 0.01	451		0.030 ± 0.02	0.004 ± 0.001
					**0.028 ± 0.003** [g]	**0.043 ± 0.001** [g]
**Selected size: De = 105 nm/Dm =77.2 nm**						
660 ± 24	0.095 ± 0.012	0.037 ± 0.004			0.014 ± 0.002	0.006 ± 0.001
665 ± 12	0.166 ± 0.013	0.024 ± 0.005		85.1	0.025 ± 0.002	0.004 ± 0.001
390 ± 6	0.071 ± 0.001	0.020 ± 0.009			0.018 ± 0.002	0.005 ± 0.001
					**0.019 ± 0.006** [g]	**0.005 ± 0.001** [g]
**Selected size: De = 147 nm/Dm = 108.1 nm**						
792 ±15	0.160 ± 0.005	0.024 ± 0.005	584		0.020 ± 0.002	0.003 ± 0.001
886 ± 19	0.172 ± 0.012	0.024 ± 0.004	500	121.9	0.02 ± 0.002	0.003 ± 0.001
1726 ± 38	0.341 ± 0.006	0.076 ± 0.003	1160		0.020 ± 0.002	0.004 ± 0.001
761 ± 12	0.174 ± 0.009	0.027 ± 0.007	498		0.022 ± 0.002	0.003 ± 0.001
					**0.02 ± 0.002** [g]	**0.003 ± 0.001** [g]
**Selected size: De = 300 nm/Dm = 220.6 nm**						
166 ± 11	0.029 ± 0.001	0.0028 ± 0.0001	204	233	0.017 ± 0.003	0.002 ± 0.001
170 ± 3	0.023 ± 0.002	0.0025 ± 0.0003	200		0.014 ± 0.001	0.002 ± 0.001
					**0.016 ± 0.002** [g]	**0.002 ± 0.001** [g]

[a]Reactant and products concentration in µM. The sample of each experiment was analyzed 3 times and the reported value represents the mean value; [b]Errors represent the standard deviation; [c]Particle mass in µg m^{-3}; [d]Median particle diameter in nm; [e]Product yield formation calculated via $\frac{[Product]}{[Reactant]} \times 100$; [f]Uncertainties derived via propagation of the concentration errors; [g]Mean value of the products concentration for each experimental condition.

Table S8: Experimental conditions and LCMS data of the size resolved measurements performed in maleic acid particles at RH = 55%. Formation yields of the products and size data are displayed.

**[Maleic acid]** [a,b]	**[Malic acid]** [a,b]	**Mass** [c]	**Median diameter** [d]	[d]**% PY_{Malic}** [e,f]
**Selected size: De = 75 nm/Dm = 48.1 nm**				
170 ± 7	0.62 ± 0.02	6.47	46.1	0.37 ± 0.02
100 ± 9	0.40 ± 0.01	6.22		0.40 ± 0.02
				**0.39 ± 0.02** [g]
**Selected size: De = 100 nm/Dm = 64.1 nm**				
124 ± 4	0.69 ± 0.08	6.64	59.4	0.57 ± 0.06
109 ± 5	0.39 ± 0.02	5.82		0.36 ± 0.02
				**0.47 ± 0.11** [g]
**Selected size: De = 150 nm/Dm = 96.2 nm**				
324 ± 13	1.02 ± 0.09	6.47	109.4	0.32 ± 0.01
188 ± 8	0.68 ± 0.02	5.55		0.36 ± 0.01
				**0.34 ± 0.02** [g]
**Selected size: De = 200 nm/Dm = 128.2 nm**				
594 ± 23	1.28 ± 0.10	57.9	131.0	0.21 ± 0.02
256 ± 10	0.48 ± 0.01	45.5		0.19 ± 0.01
				**0.20 ± 0.01** [gv]
**Selected size: De = 250 nm/Dm = 160.2 nm**				
327 ± 13	0.86 ± 0.03	80.5	174.0	0.26 ± 0.01
431 ± 17	0.97 ±0.04	68.4		0.22 ± 0.01
				**0.24 ± 0.02** [g]
**Selected size: De = 300 nm/Dm = 192.3 nm**				
402 ± 16	0.60 ±0.02	83.1	224.7	0.15 ± 0.01
401 ± 22	0.80 ± 0.02	102		0.20 ± 0.02
				**0.17 ± 0.03** [g]
**Selected size: De = 400 nm/Dm = 256.4 nm**				
693 ± 20	1.40 ± 0.15	155		0.20 ± 0.02
516 ± 20	0.73 ± 0.02	168	225.0	0.14 ± 0.01
480 ± 19	0.74 ± 0.02	76.4		0.15 ± 0.01
				**0.16 ± 0.04** [g]

[a]Reactant and products concentration in μM. The sample of each experiment was analyzed 3 times and the reported value represents the mean value; [b]Errors represent the standard deviation; [c]Particle mass in μg m^{-3}; [d]Median particle diameter in nm; [e]Product yield formation calculated via $\frac{[Product]}{[Reactant]} \times 100$; [f]Uncertainties derived via propagation of the concentration errors; [g]Mean value of the products concentration for each experimental condition.

Table S9: Experimental conditions and LCMS data of the size resolved measurements performed in aconitic acid particles at RH = 55%. Formation yields of the products and size data are displayed.

**[Aconitic acid]** [a,b]	**[Malic acid]** [a,b]	**Mass** [c]	**Median diameter** [d]	**% PY_{Malic}** [e,f]
**Selected size: De = 80 nm/Dm = 58.5 nm**				
551 ± 18	0.121 ± 0.004	1170	66.1	0.022 ± 0.003
419 ± 22	0.090 ± 0.002	1020		0.021 ± 0.040
611 ± 14	0.132 ± 0.001	1354		0.022 ± 0.020
				**0.022 ± 0.001** [g]
**Selected size: De = 100 nm/Dm = 73.1 nm**				
872 ± 54	0.124 ± 0.005	1400	82.0	0.014 ± 0.031
508 ± 11	0.120 ± 0.008	1190		0.024 ± 0.032
1110 ± 21	0.141 ± 0.006	1445		0.013 ± 0.020
				**0.017 ± 0.007** [g]
**Selected size: De = 150 nm/Dm = 109.7 nm**				
1245 ± 166	0.114 ± 0.001	6370	117.6	0.013 ± 0.002
1069 ± 101	0.099 ± 0.009	4247		0.009 ± 0.004
				**0.011 ± 0.002** [g]
**Selected size: De = 250 nm/Dm = 182.5 nm**				
2624 ± 58	0.124 ± 0.003	11940	187.7	0.005 ± 0.001
2631 ± 24	0.161 ± 0.008	13100		0.006 ± 0.001
				**0.006 ± 0.001** [g]

[a]Reactant and products concentration in µM. The sample of each experiment was analyzed 3 times and the reported value represents the mean value; [b]Errors represent the standard deviation; [c]Particle mass in µg m^{-3}; [d]Median particle diameter in nm; [e]Product yield formation calculated via $\frac{[Product]}{[Reactant]} \times 100$; [f]Uncertainties derived via propagation of the concentration errors; [g]Mean value of the products concentration for each experimental condition.

Table S10: DFT Energies of important species involved in the CA reactions calculated at the ωB97X-V/TZVPPD level.

**Species**	**Energy (hartree)**
OH	-75.740528
O_2	-150.339628
H_2O	-76.437115
HO_2	-150.924155
CO_2	-188.60535
Citric acid	-760.174423
CA middle decarboxylation product (Ra)	-570.920538
CA end decarboxylation product (Rb)	-570.902452
CA H abstraction product (Rc)	-759.508424
Ra-O radical	-646.145152
Rb-O radical	-646.11348
Rc-O radical	-834.7114
oxyglutaric acid P2	-570.363398
malonic acid	-417.709451
glyoxylic acid	-303.11131
CH_2COOH	-228.446148
Rb aldehyde product P3	-645.589973
hydroxy maleic acid	-531.032763
hydroxy maleic acid radical	-531.617665
formaldehyde	-114.511807
malic acid	-532.26278

Table S11: DFT Energies of important species involved in the MA reactions calculated at the ωB97X-V/TZVPPD level.

**Species**	**Energy (hartree)**
OH	-75.740528
HO_2	-150.924155
H_2O	-76.437115
O_2	-150.339628
CO_2	-188.60535
Maleic Acid	-455.790172
malic acid pathway	
Malic Acid	-532.26278
Malic Acid Radical	-531.587834
TS for H-abstraction from H2O	-607.989877
acrylic acid pathway	
Carboxy radical	-455.097903
cis Acrylic Acid Radical	-266.501669
Acrylic Acid	-267.193667
TS1: COO-H abstraction	-531.527914
TS2: R-COO bond cleavage	-455.088959
TS3: H-abstraction from H_2O	-342.928307

REFERENCES AND NOTES

1. J. H. Seinfeld, S. N. Pandis, *Atmospheric Chemistry and Physics: From Air Pollution to Climate Change* (John Wiley and Sons, 1998).

2. M. Kanakidou, J. H. Seinfeld, S. N. Pandis, I. Barnes, F. J. Dentener, M. C. Facchini, R. V. Dingenen, B. Ervens, A. Nenes, C. J. Nielsen, E. Swietlicki, J. P. Putaud, Y. Balkanski, S. Fuzzi, J. Horth, G. K. Moortgat, R. Winterhalter, C. E. L. Myhre, K. Tsigaridis, E. Vignati, E. G. Stephanou, J. Wilson, Organic aerosol and global climate modelling: A review. *Atmos. Chem. Phys.* **5**, 1053–1123 (2005).

3. U. Pöschl, Atmospheric aerosols: Composition, transformation, climate and health effects. *Angew. Chem. Int. Ed.* **44**, 7520–7540 (2005).

4. C. George, M. Ammann, B. D'Anna, D. J. Donaldson, S. A. Nizkorodov, Heterogeneous photochemistry in the atmosphere. *Chem. Rev.* **115**, 4218–4258 (2015).

5. C. D. Cappa, D. L. Che, S. H. Kessler, J. H. Kroll, K. R. Wilson, Variations in organic aerosol optical and hygroscopic properties upon heterogeneous OH oxidation. *J. Geophys. Res.* **116**, D15204 (2011).

6. I. J. George, A. Vlasenko, J. G. Slowik, K. Broekhuizen, J. P. D. Abbatt, Heterogeneous oxidation of saturated organic aerosols by hydroxyl radicals: Uptake kinetics, condensed-phase products, and particle size change. *Atmos. Chem. Phys.* **7**, 4187–4201 (2007).

7. M. J. Molina, A. V. Ivanov, S. Trakhtenberg, L. T. Molina, Atmospheric evolution of organic aerosol. *Geophys. Res. Lett.* **31**, L22104 (2004).

8. J. Li, D. A. Knopf, Representation of multiphase OH oxidation of amorphous organic aerosol for tropospheric conditions. *Environ. Sci. Technol.* **55**, 7266–7275 (2021).

9. J. Lelieveld, S. Gromov, A. Pozzer, D. Taraborrelli, Global tropospheric hydroxyl distribution, budget and reactivity. *Atmos. Chem. Phys.* **16**, 12477–12493 (2016).

10. J. K. Lee, K. L. Walker, H. S. Han, J. Kang, F. B. Prinz, R. M. Waymouth, H. G. Nam, R. N. Zare, Spontaneous generation of hydrogen peroxide from aqueous microdroplets. *Proc. Natl. Acad. Sci. U.S.A.* **116**, 19294–19298 (2019).

11. D. Nguyen, S. C. Nguyen, Revisiting the effect of the air–water interface of ultrasonically atomized water microdroplets on H_2O_2 formation. *J. Phys. Chem. B* **126**, 3180–3185 (2022).

12. K. Li, Y. Guo, S. A. Nizkorodov, Y. Rudich, M. Angelaki, X. Wang, T. An, S. Perrier, C. George, Spontaneous dark formation of OH radicals at the interface of aqueous atmospheric droplets. *Proc. Natl. Acad. Sci. U.S.A.* **120**, e2220228120 (2023).

13. M. Angelaki, Y. Carreira Mendes Da Silva, S. Perrier, C. George, Quantification and mechanistic investigation of the spontaneous H_2O_2 generation at the interfaces of salt-containing aqueous droplets. *J. Am. Chem. Soc.* **146**, 8327–8334 (2024).

14. J. K. Lee, H. S. Han, S. Chaikasetsin, D. P. Marron, R. M. Waymouth, F. B. Prinz, R. N. Zare, Condensing water vapor to droplets generates hydrogen peroxide. *Proc. Natl. Acad. Sci. U.S.A.* **117**, 30934–30941 (2020).

15. J. P. Heindel, H. Hao, R. A. LaCour, T. Head-Gordon, Spontaneous formation of hydrogen peroxide in water microdroplets. *J. Phys. Chem. Lett.* **13**, 10035–10041 (2022).

16. M. A. Mehrgardi, M. Mofidfar, R. N. Zare, Sprayed water microdroplets are able to generate hydrogen peroxide spontaneously. *J. Am. Chem. Soc.* **144**, 7606–7609 (2022).

17. M. Angelaki, J. d'Erceville, D. J. Donaldson, C. George, pH affects the spontaneous formation of H_2O_2 at the air–water interfaces. *J. Am. Chem. Soc.* **146**, 25889–25893 (2024).

18. P. Jungwirth, D. J. Tobias, Specific ion effects at the air/water interface. *Chem. Rev.* **106**, 1259–1281 (2006).

19. H. Xiong, J. K. Lee, R. N. Zare, W. Min, Strong electric field observed at the interface of aqueous microdroplets. *J. Phys. Chem. Lett.* **11**, 7423–7428 (2020).

20. H. Hao, I. Leven, T. Head-Gordon, Can electric fields drive chemistry for an aqueous microdroplet? *Nat. Commun.* **13**, 280 (2022).

21. J. K. Lee, D. Samanta, H. G. Nam, R. N. Zare, Micron-sized water droplets induce spontaneous reduction. *J. Am. Chem. Soc.* **141**, 10585–10589 (2019).

22. J. Jung, K. Kawamura, Enhanced concentrations of citric acid in spring aerosols collected at the Gosan background site in East Asia. *Atmos. Environ.* **45**, 5266–5272 (2011).

23. S. K. R. Boreddy, P. Hegde, A. R. Aswini, Summertime high abundances of succinic, citric, and glyoxylic acids in Antarctic aerosols: Implications to secondary organic aerosol formation. *JGR Atmos.* **127**, e2021JD036172 (2022).

24. E. Barbaro, M. Feltracco, F. De Blasi, C. Turetta, M. Radaelli, W. Cairns, G. Cozzi, G. Mazzi, M. Casula, J. Gabrieli, C. Barbante, A. Gambaro, Chemical characterization of atmospheric aerosols at a high-altitude mountain site: A study of source apportionment. *Atmos. Chem. Phys.* **24**, 2821–2835 (2024).

25. K. Sato, F. Ikemori, S. Ramasamy, A. Fushimi, K. Kumagai, A. Iijima, Y. Morino, Four- and five-carbon dicarboxylic acids present in secondary organic aerosol produced from anthropogenic and biogenic volatile organic compounds. *Atmos.* **12**, 1703 (2021).

26. J. F. Davies, K. R. Wilson, Nanoscale interfacial gradients formed by the reactive uptake of OH radicals onto viscous aerosol surfaces. *Chem. Sci.* **6**, 7020–7027 (2015).

27. C. S. Sheldon, J. M. Choczynski, K. Morton, T. Palacios Diaz, R. D. Davis, J. F. Davies, Exploring the hygroscopicity, water diffusivity, and viscosity of organic–inorganic aerosols – A case study on internally-mixed citric acid and ammonium sulfate particles. *Environ. Sci. Atmos.* **3**, 24–34 (2023).

28. C. Peng, M. N. Chan, C. K. Chan, The hygroscopic properties of dicarboxylic and multifunctional acids: Measurements and UNIFAC predictions. *Environ. Sci. Technol.* **35**, 4495–4501 (2001).

29. S. Han, J. Hong, Q. Luo, H. Xu, H. Tan, Q. Wang, J. Tao, Y. Zhou, L. Peng, Y. He, J. Shi, N. Ma, Y. Cheng, H. Su, Hygroscopicity of organic compounds as a function of organic functionality, water solubility, molecular weight, and oxidation level. *Atmos. Chem. Phys.* **22**, 3985–4004 (2022).

30. A. K. Salameh, L. J. Mauer, L. S. Taylor, Deliquescence lowering in food ingredient mixtures. *J. Food Sci.* **71**, E10–E16 (2006).

31. H. Veith, C. Luebbert, G. Sadowski, Predicting deliquescence relative humidities of crystals and crystal mixtures. *Molecules* **26**, 3176 (2021).

32. C. Peng, L. Chen, M. Tang, A database for deliquescence and efflorescence relative humidities of compounds with atmospheric relevance. *Fundam. Res.* **2**, 578–587 (2022).

33. M. Girod, E. Moyano, D. I. Campbell, R. G. Cooks, Accelerated bimolecular reactions in microdroplets studied by desorption electrospray ionization mass spectrometry. *Chem. Sci.* **2**, 501–510 (2011).

34. B. M. Marsh, K. Iyer, R. G. Cooks, Reaction acceleration in electrospray droplets: Size, distance, and surfactant effects. *J. Am. Soc. Mass Spectrom.* **30**, 2022–2030 (2019).

35. X. Yan, R. M. Bain, R. G. Cooks, Organic reactions in microdroplets: Reaction acceleration revealed by mass spectrometry. *Angew. Chem. Int. Ed.* **55**, 12960–12972 (2016).

36. Z. Wei, Y. Li, R. G. Cooks, X. Yan, Accelerated reaction kinetics in microdroplets: Overview and recent developments. *Annu. Rev. Phys. Chem.* **71**, 31–51 (2020).

37. J. K. Lee, S. Kim, H. G. Nam, R. N. Zare, Microdroplet fusion mass spectrometry for fast reaction kinetics. *Proc. Natl. Acad. Sci. U.S.A.* **112**, 3898–3903 (2015).

38. D. E. Cabelli, B. H. J. Bielski, A pulse radiolysis study of some dicarboxylic acids of the citric acid cycle. The kinetics and spectral properties of the free radicals formed by reaction with the OH radical. *Z. Naturforsch. B* **40**, 1731–1737 (1985).

39. G. E. Adams, J. W. Boag, J. Currant, B. D. Michael, Absolute rate constants for the reaction of the hydroxyl radical with organic compounds. *Pulse Radiolysis* , 131–143 (1965).

40. J. P. Heindel, R. A. LaCour, T. Head-Gordon, The role of charge in microdroplet redox chemistry. *Nat. Commun.* **15**, 3670 (2024).

41. A. J. Colussi, Mechanism of hydrogen peroxide formation on sprayed water microdroplets. *J. Am. Chem. Soc.* **145**, 16315–16317 (2023).

42. M. Riva, V. Pospisilova, C. Frege, S. Perrier, P. Bansal, S. Jorga, P. Sturm, J. A. Thornton, U. Rohner, F. Lopez-Hilfiker, Evaluation of a reduced-pressure chemical ion reactor utilizing adduct ionization for the detection of gaseous organic and inorganic species. *Atmos. Meas. Tech.* **17**, 5887–5901 (2024).

43. L. Gao, I. Zgheib, E. Stergiou, F. Sari Doré, C. Carstens, M. Dupanloup, F. Bourgain, S. Perrier, M. Riva, Characterization of the newly designed wall-free particle evaporator (WALL-E) for online measurements of atmospheric particles. EGUsphere [Preprint], https://doi.org/10.5194/egusphere-2025-1072 (2025).

44. C. Corvaja, P. L. Nordio, G. Giacometti, Free radicals from citric acid. *Trans. Faraday Soc.* **62**, 3400 (1966).

45. A. Gallo Jr., N. H. Musskopf, X. Liu, Z. Yang, J. Petry, P. Zhang, S. Thoroddsen, H. Im, H. Mishra, On the formation of hydrogen peroxide in water microdroplets. *Chem. Sci.* **13**, 2574–2583 (2022).

46. M. Mofidfar, M. A. Mehrgardi, Y. Xia, R. N. Zare, Dependence on relative humidity in the formation of reactive oxygen species in water droplets. *Proc. Natl. Acad. Sci. U.S.A.* **121**, e2315940121 (2024).

47. Q. Z. Rasool, M. Shrivastava, Y. Liu, B. Gaudet, B. Zhao, Modeling the impact of the organic aerosol phase state on multiphase OH reactive uptake kinetics and the resultant heterogeneous oxidation timescale of organic aerosol in the amazon rainforest. *ACS Earth Space Chem.* **7**, 1009–1024 (2023).

48. X. Wang, N. Hayeck, M. Brüggemann, L. Abis, M. Riva, Y. Lu, B. Wang, J. Chen, C. George, L. Wang, Chemical characteristics and brown carbon chromophores of atmospheric organic aerosols over the yangtze river channel: A cruise campaign. *JGR Atmospheres* **125**, e2020FD032497 (2020).

49. F. Zhao, J. Qian, F. Quan, C. Wu, Y. Zheng, L. Zhou, Aconitic acid derived carbon dots as recyclable "on–off–on" fluorescent nanoprobes for sensitive detection of mercury(II) ions, cysteine and cellular imaging. *RSC Adv.* **7**, 44178–44185 (2017).

50. M. Uchimiya, J. E. Knoll, Accumulation of carboxylate and aromatic fluorophores by a pest-resistant sweet sorghum [*Sorghum bicolor* (L.) Moench] genotype. *ACS Omega* **4**, 20519–20529 (2019).

51. Mariam, M. Joshi, P. Khandare, A. Koli, A. Khan, B. K. Sapra, Influence of sheath air humidity on measurement of particle size distribution by scanning mobility particle sizer. *J. Aerosol Sci.* **111**, 18–25 (2017).

52. L. H. Yang, M. Takeuchi, Y. Chen, N. L. Ng, Characterization of thermal decomposition of oxygenated organic compounds in FIGAERO-CIMS. *Aerosol Sci. Tech.* **55**, 1321–1342 (2021).

53. Y. Shao, L. F. Molnar, Y. Jung, J. Kussmann, C. Ochsenfeld, S. T. Brown, A. T. B. Gilbert, L. V. Slipchenko, S. V. Levchenko, D. P. O'Neill, R. A. Di Stasio Jr., R. C. Lochan, T. Wang, G. J. O. Beran, N. A. Besley, J. M. Herbert, C. Y. Lin, V. T. Voorhis, S. H. Chien, A. Sodt, R. P. Steele, V. A. Rassolov, P. E. Maslen, P. P. Korambath, R. D. Adamson, B. Austin, J. Baker, E. F. C. Byrd, H. Dachsel, R. J. Doerksen, A. Dreuw, B. D. Dunietz, A. D. Dutoi, T. R. Furlani, S. R. Gwaltney, A. Heyden, S. Hirata, C.-P. Hsu, G. Kedziora, R. Z. Khalliulin, P. Klunzinger, A. M. Lee, M. S. Lee, W. Z. Liang, I. Lotan, N. Nair, B. Peters, E. I. Proynov, P. A. Pieniazek, Y. M. Rhee, J. Ritchie, E. Rosta, C. D. Sherrill, A. C. Simmonett, J. E. Subotnik, H. L. Woodcock III, W. Zhang, A. T. Bell, A. K. Chakraborty, D. M. Chipman, F. J. Keil, A. Warshel, W. J. Hehre, H. F. Schaefer, J. Kong, A. I. Krylov, P. M. W. Gill, M. Head-Gordon, Advances in Methods and Algorithms in a Modern Quantum Chemistry Program Package, *Phys. Chem. Chem. Phys.* **8**, 3172 (2006).
